# Nervisides I–J: Unconventional Side-Chain-Bearing Cycloartane Glycosides from *Nervilia concolor*

**DOI:** 10.3390/molecules24142599

**Published:** 2019-07-17

**Authors:** Thi-Ngoc-Mai Tran, Guillaume Bernadat, Dinh-Tri Mai, Van-Kieu Nguyen, Jirapast Sichaem, Tan-Phat Nguyen, Cong-Luan Tran, Phuong-Vy Do, Nguyen-Minh-An Tran, Huu-Hung Nguyen, Mehdi A. Beniddir, Thuc-Huy Duong, Pierre Le Pogam

**Affiliations:** 1 Graduate University of Science and Technology, Vietnam Academy of Science and Technology, 18 Hoang Quoc Viet, Cau Giay, Ha Noi 100803, Vietnam; 2 Institute of Applied Sciences, Ho Chi Minh City University of Technology (HUTECH), 475 A Dien Bien Phu Street, Ward 25, Binh Thanh District, Ho Chi Minh City 758307, Viet Nam; 3 Équipe “Pharmacognosie–Chimie des Substances Naturelles”, BioCIS, Univ. Paris-Sud, CNRS, Université Paris-Saclay, 5 Rue Jean-Baptiste Clément, 92290 Châtenay-Malabry, France; 4 Institute of Chemical Technology, Vietnam Academy of Science and Technology, 01 Mac Dinh Chi, Ho Chi Minh City 758307, Vietnam; 5 Center of Excellence in Natural Products Chemistry, Department of Chemistry, Faculty of Science, Chulalongkorn University, Pathumwan, Bangkok 10330, Thailand; 6 Faculty of Science and Technology, Thammasat University Lampang Campus, Lampang 52190, Thailand; 7 Mien Dong University of Technology, MUT, Dong Nai Provine 813847, Vietnam; 8 Ho Chi Minh City University of Technology (HCMUT), Ho Chi Minh City 758307, Vietnam; 9 Industrial University of Ho Chi Minh City, Ho Chi Minh City 758307, Vietnam; 10 Faculty of Biotechnology, Nguyen Tat Thanh University, 300A Nguyen Tat Thanh Str., Dist. 4, Ho Chi Minh City 758307, Vietnam; 11 Department for Management of Science and Technology Development, Ton Duc Thang University, Ho Chi Minh City 758307, Vietnam; 12 Faculty of Applied Sciences, Ton Duc Thang University, Ho Chi Minh City 758307, Vietnam

**Keywords:** *Nervilia concolor*, triterpene, saponoside, cycloartane, xylopyranose

## Abstract

Two new cycloartane glycosides, nervisides I–J, were isolated from *Nervilia concolor* whole plants. Their structures were unambiguously established by interpretation of their HRESIMS and 1D and 2D NMR data. These cycloartanes comprised a stereogenic center at C-24, the *R* configuration of which was assigned based on DFT-NMR calculations and the subsequent DP4 probability score. These compounds were tested for cytotoxicity against K562 and MCF-7 tumor cell lines, revealing mild cytotoxic activity.

## 1. Introduction

The terrestrial orchid genus *Nervilia* contains approximately 65 species which are mostly found in tropical and subtropical Africa, Asia, Australia, and the Southwest Pacific Islands [1]. The herbal plant *Nervilia concolor* (Blume) Schltr. (Orchidaceae) (syn. *N. aragoana*) is regionally distributed in Dak Lak, KonTum, An Giang, and Dong Nai provinces of Vietnam. This plant is widely used in traditional Chinese medicine for a variety of diseases, such as bronchitis, stomatitis, acute pneumonia, and laryngitis [2,3,4,5,6]. As of 2019, phytochemical studies undertaken on *Nervilia* species have led to the identification of ca. 60 compounds, mostly including flavonoids (>20), a dozen terpenes, and some sterols and amino acids. Nevertheless, as far as can be ascertained, *N. concolor* (syn. *N. aragoana*) has not been studied from a chemical perspective so far. This article describes the isolation and structural elucidation of two new cycloartane glycosides, namely, nervisides I–J (**1**–**2**), from this plant source. Consistent with previously reported structures of *Nervilia* species, these natural products reveal an unconventional side chain bearing an alkyl substituent at C-24, the absolute configuration of which has been difficult to assign, often leading to an undetermined configuration of this stereogenic center in structurally related compounds, including nervisides A–H isolated from *Nervilia fordii* [7,8], which were not defined as to this carbon. Such C-24 hydroxymethylated cycloartanes were also repeatedly reported to occur within *Passiflora* species [9,10,11]. In this study, besides benefitting from an extensive set of NMR and HRESIMS analyses, C-24 configuration was determined based on GIAO NMR shift calculation of the two possible epimers and the subsequent DP4 probability score, leading to the assignment of a *24R* configuration with a quantifiable confidence of 99.2%.

## 2. Results and Discussion

**Figure 1:**
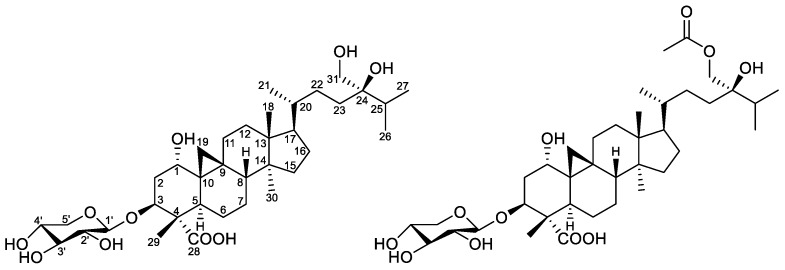
Structures of compounds **1–2**.

**Figure 2:**
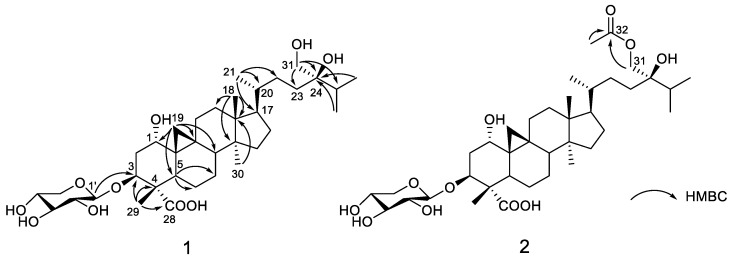
Key HMBC correlations of **1–2**.

**Figure 3:**
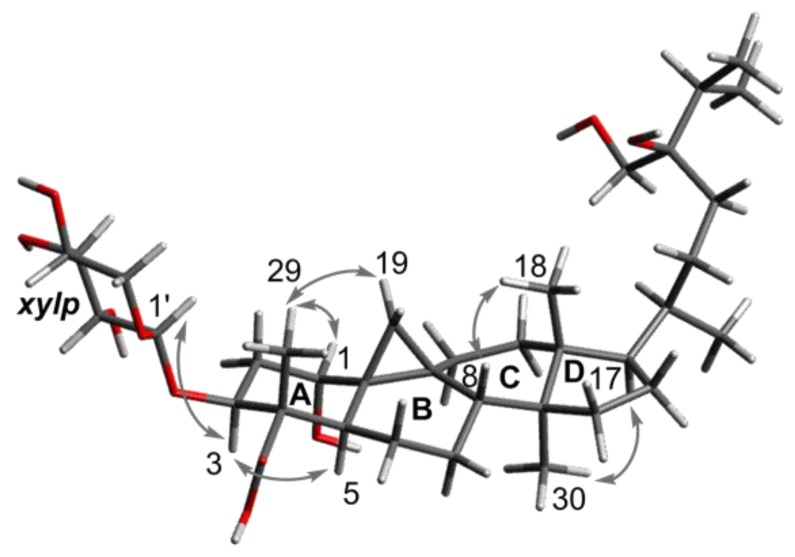
Key NOE correlations of **1**.

Compound **1** was obtained as a white gum. Its molecular formula was determined to be C_36_H_60_O_10_ based on the deprotonated molecular ion at *m*/*z* 651.4103 (calcd for C_36_H_59_O_10_, 651.4114). The ^13^C NMR spectrum, in conjunction with the HSQC spectrum, revealed 36 carbon signals, of which 31 could be assigned to a triterpenoid sapogenol core and 5 belonged to a monosaccharide unit. The 31 carbon resonances of the aglycone part consisted of 6 methyl carbons; 11 methylene carbons, 1 of which was oxygenated; 8 methine carbons, 3 of which bore oxygen functionalities; and 7 quaternary carbons comprising 1 carbonyl and an oxygenated carbon. Both the ^1^H upfield methylenic protons at δ_H_ 0.36 and 0.56 (each 1H, doublet with *J* = 4.0 Hz) and the six unsaturation degrees of the aglycone moiety led to define a carboxylic-acid-substituted cycloartane scaffold [12,13] (Figure 1 and Supplementary Materials). The thorough analysis of the COSY, HSQC, and HMBC spectra led to fully assign the ^1^H and ^13^C signals for compound **1** (Table 1). In the A-ring, a methyl and a carboxylic acid group could be assigned at C-4 (δ_C_ 53.0) based on HMBC correlations of both oxymethine H-3 (δ_H_ 4.40) and methyl H_3_-29 (δ_H_ 0.97) to carbons C-4 and C-28 (δ_C_ 178.5) as well as the HMBC cross-peak of H_3_-29 and C-3 (δ_C_ 78.8) (Figure 2). A hydroxy group could be anchored at C-1 based on HMBC cross-peaks of H_2_-19, H-3, and H-5 to C-1 and of H-1 (δ_H_ 3.34) to C-2 (δ_C_ 36.1), C-3 (δ_C_ 78.8), and C-5 (δ_C_ 36.6). A deshielded signal assigned to H-1 eq., partly overlapped with the water signal, resonated at 3.34 ppm as a broad singlet, diagnostic of the occurrence of a α-hydroxy group owing to the lack of a trans-diaxial coupling constant [14]. The H-3 signal appeared as a double doublet owing to diaxial (*J* = 12.0 Hz) and axial–equatorial coupling (*J* = 4.5 Hz) defining its axial orientation [9,15,16]. The glycosylation shift at C-3 (δ_C_ 78.8) of the aglycone indicated that the monosaccharide was linked at this specific position, as further backed up by the long-range heteronuclear correlation from H-1′ to C-3. The δ 2.5–4.5 ppm region of the ^1^H NMR spectrum validated the occurrence of a single saccharide, which could be directly identified as a xylopyranose unit based on the diagnostic triplet signal for the H-5′ α-proton at 2.97 ppm [14]. The COSY spectrum revealed the correlations of all the protons in the xylopyranose ring, and the magnitude of the vicinal coupling constant values were in excellent agreement with formerly reported J values for β-d-xylopyranose residues [17,18,19]. The NOE cross-peaks between H-1, H_2_-19, and H_3_-29 defined their β-orientation, thereby determining the α-position of the 4-COOH group (Figure 3). The canonical stereochemistry of the ABCD rings [20] was supported by the nearly identical ^1^H and ^13^C NMR data of **1** with cycloartane triterpenes [21], nervisides A–C [7], nervisides D–H [8], and cyclopassifloic acid series [10], as supported by the key NOE correlations outlined in Figure 3. This only left the relative configuration of the side chain pending assignment. The HMBC experiment revealed correlations between the oxygenated methylene protons resonating at δ_H_ 3.25 to C-23, C-24, and C-25 that defined the occurrence of a hydroxymethyl group at C-24 consistently with the side chain of formerly reported nervisides. Accordingly, nonconventional side chain triterpenes and sterols were repeatedly described from *Nervilia* species. [22,23]. Defining the absolute configuration of C-24 alkyl sterols and triterpenes is a vexing problem in NMR spectroscopy that has tentatively been overcome through tailored chromatographic procedures [24,25]. These difficulties result in some authors not defining C-24 absolute configuration on such related scaffolds including cyclotricuspidosides A–C [26] and nervisides A–H [7,8], even though derivatization-based NMR spectroscopy affords reliable outcomes as to this specific point [9,10,27]. To assign the absolute configuration at C-24, ^13^C NMR chemical shift calculations of simplified bicyclic models only including cycles C and D were performed using electronic structure methods of the lowest-energy conformer of both C-24 epimers. The spectral position of triterpene carbon side-chain bands does not vary over extensive sets of derivatives involving the central ring system [28]. In particular, chemical shifts of the side chain could be used to determine the absolute configuration of C-24 in several sterols owing to their chemical shifts being insensitive to structural changes remote from the asymmetric carbon [29]. The lowest energy conformation of the core ring system was more quickly located by seeding the potential energy surface scan with initial coordinates available in X-ray crystallographic CIF files associated with formerly reported cycloartanes [30,31,32]. Subsequent ^13^C NMR data comparison of the two possible epimers against the experimental dataset resulted in the prediction of the *24R* configuration with a quantifiable confidence of 99.2%. Accordingly, compound **1**, namely nerviside I, was identified as 3β-*O*-d-xylopyranosyl-1α,24R,31-trihydroxylcycloartan-28-oic acid.

Compound **2**, obtained as a white amorphous solid, gave a molecular formula of C_38_H_62_O_11_ based on its negative-ion mode HRESIMS data, which displayed a [M–H]^−^ peak at *m*/*z* 693.4215 (calcd for C_38_H_61_O_11_, 693.4219). This hinted that **2** differed from **1** by a supplementary acetyl group. Accordingly, both the ^1^H and ^13^C NMR spectroscopic data were very similar between the two compounds, but the ^1^H NMR spectrum of **2** revealed one more methyl group resonating at δ_H_ 1.99 (3H, s), while the ^13^C NMR spectrum exhibited one more carbonyl carbon at δ_C_ 170.3. The occurence of an acetyl group was deduced from the HMBC correlation originating from the methyl proton at δ_H_ 1.99 to carbonyl carbon C-32 at δ_C_ 170.3. The thorough analysis of the 2D NMR spectra determined a similar cycloartane glycoside core as in compound **1** except for the acetylation of the hydroxy group at C-31, further backed up by the key HMBC correlation from H_2_-31 (3.88 and 3.84) to C-32. An identical C-24 R configuration was assigned based on the good agreement between the carbon signals due to C-23, C-24, and C-25 and biogenetic considerations. From the above evidence, compound **2,** namely nerviside J, was established as 3β-*O*-d-xylopyranosyl-31-*O*-acetyl-1α,24R-dihydroxycycloartan-28-oic acid.

In this study, compounds **1**–**2** were evaluated for their cytotoxicity against K562 (chronic myelogenous leukemia) and MCF-7 (breast cancer) cell lines. Both compounds **1** and **2** exerted moderate activity against these two cancer cell lines, with respective IC_50_ values of 20.5 (±0.2) and 20.6 (±0.1) µg/mL for **1** and 40.1 (±0.6) and 90.5 (±3.5) µg/mL for **2**.

## 3. Materials and Methods

### 3.1. General

NMR spectra were performed on a Bruker AM500 FT-NMR spectrometer (500 MHz for ^1^H NMR and 125 MHz for ^13^C NMR). The ESI-HRMS data were generated with a Bruker MicroTOF-QII spectrometer (Bremen, Germany). Open-column chromatography was performed on silica gel 40–63 µm phase (Merck, Darmstadt, Germany) and reversed-phase C_18_ (Merck, Darmstadt, Germany). TLC analyses were carried out on precoated silica gel 60 F_254_ (Merck, Darmstadt, Germany), and spots were visualized by spraying the plates with 10% H_2_SO_4_ solution followed by heating.

### 3.2. Plant Material

*N. concolor* whole plants were collected in the Cu M’gar district, Dak Lak province, from August to November 2017 and authenticated by Dr. Cong-Luan Tran, Research Center of Ginseng and Medicinal Materials of Ho Chi Minh City National Institute of Medicinal Materials. A voucher specimen (no. NA-0621) was deposited in the Bioactive Compounds Laboratory, Institute of Chemical Technology.

### 3.3. Extraction and Isolation

The dried whole plants (4.0 kg) were milled prior to being extracted with 96% EtOH three times (3 × 30 L, each 8 h) at room temperature. The filtered solution was concentrated in vacuo to afford a crude extract (280 g). This dried residue was successively re-extracted using solvents of increasing polarities: *n*-hexane (H, 110 g), CHCl_3_ (C, 25 g), EtOAc (EA, 90 g), and H_2_O (W, 45 g). Extract EA was subjected to silica gel column chromatography and eluted with a chloroform/MeOH solvent system (stepwise, 1:0 to 1:0) to afford seven fractions: E1–E7. Fraction E5 (20 g) was selected for further purification using column chromatography based on a CHCl_3_–MeOH solvent system gradient (20:1 to 1:1) to yield five subfractions (E5.1–5.5). Subfraction E5.1 (1.1 g) was subjected to silica gel column chromatography using an isocratic mobile phase consisting of a CHCl_3_/MeOH/H_2_O solvent system (10:1:0.1) to afford 1 (10 mg) and 2 (14 mg).

*Nerviside I (***1**). White gum. ^1^H- and ^13^C NMR see Table 1; HRESIMS *m/z* 651.4103 [M–H]^−^ (calcd for C_36_H_59_O_10_, 651.4114).

*Nerviside J* (**2**). White amorphous solid ^1^H- and ^13^C NMR see Table 1; HRESIMS *m/z* 693.4215 [M–H]^−^ (calcd for C_38_H_61_O_11_^−^, 693.4219).

### 3.4. Computational Chemistry

Truncated models of 1 (without the sugar moiety) and its epimer were assembled and the cycloartane skeleton was arranged in both with a conformation identical to that found in [30,31,32]. A conformation search was then performed on the overall structure using the basin hopping method [33] with the MMFF94 force field [34] as implemented in the *scan* program from the Tinker v8.6.1 software package [35,36,37]. Coordinates of the lowest energy minimum for both epimers were then further truncated by keeping only rings C and D and optimized at the B3LYP/6-31G(d) level [38,39,40] using the Gaussian 16 software package [41]. Vibrational analysis within the harmonic approximation was performed at the same level of theory upon geometrical optimization convergence prior to characterizing local minima by the absence of imaginary frequency. Chemical shifts were deduced from NMR shielding tensors calculated using the GIAO method [42,43] and corrected against values for the corresponding nucleus in TMS, both at the same level of theory. DP4 probability values were calculated using online implementation available from http://www-jmg.ch.cam.ac.uk/tools/nmr/DP4/ [44].

### 3.5. Biological Assays

Cytotoxic activities of the formerly unreported metabolites were evaluated against the MCF-7 (breast cancer) and K562 (chronic myelogenous leukemia) tumor cell lines. These two cell lines were cultured in RPMI 1640 medium or in DMEM medium, respectively; supplemented with 10% fetal bovine serum (FBS), 100 IU/mL penicillin, and 100 μg/mL streptomycin; and maintained at 37 °C and 5% CO_2_ with 95% humidity. Viable cells were counted and inoculated in a 96-well plate with a density of 10^4^ cells/100 μL/well for MCF-7 and 10^5^ cells/100 μL/well for K562. After 24 h, the cells were treated with the compounds and doxorubicin (positive control) diluted in culture media at 100, 50, 25, 12.5, 6.25, 3.125, and 0 µg/mL concentration containing 1%, 0.5%, 0.25%, 0.125%, 0.0625%, 0.03125%, and 0% dimethyl sulfoxide (DMSO), respectively. DMSO in culture media was used as a negative control. In addition, culture medium without cells was used as a blank. All experiments were done in triplicate. The plates were incubated in 5% CO_2_ with 95% humidity at 37 °C for 72 h. Ten microliters of 3-(4,5-dimethylthiazol-2-yl)-2,5-diphenyltetrazolium bromide (MTT, 5 mg/mL stock solution) were added to each well and incubated in 37 °C in 5% CO_2_ for 3.5 h. Seventy microliters of detergent reagent (10% SDS) were added to each well and the plate was maintained in 37 °C for 16 h. The optical density of each well was read by using a scanning multiwall spectrophotometer (Sunrise) at a wavelength of 595 nm. Cell survival was measured as the percentage absorbance compared to the negative control (DMSO-treated cells).

## 4. Conclusions

The ethnopharmacological relevance of *N. concolor* paved the way for the currently reported phytochemical investigation that resulted in the isolation and structural elucidation of two new C-24 alkyl-substituted cycloartane glycosides, namely, nervisides I–J. While unconventional side-chain-bearing triterpenes and sterols have regularly been reported from *Nervilia* species, these compounds are the first nervisides having a defined C-24 absolute configuration, deduced from a DP4-based computational chemistry approach.

## Figures and Tables

**Table 1:** ^13^C and ^1^H NMR spectroscopic data (125/500 MHz) for **1**–**2** in dimethyl sulfoxide (DMSO)-d_6_ (δ in ppm).

	1		2	
	δ_C_	δ_H_ (*J*, Hz)	δ_C_	δ_H_ (*J*, Hz)
1	70.8	3.34, 1H, br s	70.7	3.34, 1H, br s
2	36.1	1.84, 1H, m 1.60, 1H, m	36.0	1.84, 1H, m 1.59, 1H, m
3	78.8	4.40, 1H, dd, 12.0, 4.5	78.8	4.40, 1H, dd, 12.0, 4.5
4	53.0	_	52.9	_
5	36.6	2.41, 1H, dd, 13.0, 4.5	36.5	2.42,1H, dd, 12.5, 3.5
6	22.0	1.14, 1H, m 0.84, 1H, m	21.9	1.16, 1H, m 0.83, 1H, m
7	25.1	1.02, 2H, m	25.0	1.02, 2H, m
8	47.5	1.43, 1H, m	47.3	1.46, 1H, m
9	20.1	-	19.9	-
10	28.9	-	28.7	
11	25.0	2.27, 1H, m 1.17, 1H, m	24.9	2.27, 1H, m 1.19, 1H, m
12	32.6	1.55–1.57, 2H, m	32.8	1.56–1.60, 2H, m
13	44.8	-	44.7	-
14	48.7	-	48.6	-
15	35.3	1.21–1.22, 2H, m	35.2	1.26–1.29, 2H, m
16	27.8	1.84, 1H, m 1.22, 1H, m	27.6	1.84, 1H, m 1.23, 1H, m
17	51.9	1.54, 1H, m	51.6	1.57, 1H, m
18	18.0	0.91, 3H, s	17.8	0.90, 3H, m
19	28.8	0.36, 1H, d, 4.0 0.56, 1H, d, 4.0	28.7	0.35, 1H, d, 4.0 0.57, 1H, d, 4.0
20	36.4	1.26, 1H, m	36.0	1.29, 1H, m
21	18.3	0.82, 3H, s	18.2	0.85, 3H, s
22	29.0	1.43, 1H, m 0.92, 1H, m	29.0	1.45, 1H, m 1.00, 1H, m
23	30.8	1.43, 1H, m 1.23, 1H, m	30.9	1.45, 1H, m 1.30, 1H, m
24	74.6	-	73.4	_
25	32.3	1.70, 1H, m	32.4	1.72, 1H, m
26	17.1	0.80–0.82, 3H, m	16.8	0.80–0.85, 3H, m
27	17.1	0.80–0.82, 3H, m	16.7	0.80–0.85, 3H, m
28	178.5		178.6	
29	9.5	0.97, 3H, s	9.5	0.97, 3H, s
30	19.1	0.89, 3H, s	19.0	0.91, 3H, s
31	64.7	3.25, 2H, m	66.9	3.89, 1H, d, 11.0 3.85, 2H, d, 11.0
1’	104.1	4.14, 1H, d, 7.5	104.0	4.15, 1H, d, 7.5
2’	73.7	2.88, 1H, dd, 9.0, 7.5	73.5	2.88, 1H, t, 8.5
3’	76.4	3.05, 1H, t, 9.0	76.3	3.06, 1H, t, 8.5
4’	69.6	3.23, 1H, m	69.5	3.24, 1H, m
5’	65.7	3.66, 1H, dd, 11.5, 5.0	65.6	3.66, 1H, dd, 11.0, 5.0
		2.97, 1H, t, 11.5		2.97, 1H, t, 11.0
OAc			20.7	1.99, 3H, s
			170.3	

## Supplementary Materials

The following are available online. ^1^H, ^13^C NMR, HMBC, and HRMS spectra for **1**–**2**, DFT calculations results for 24*R* and 24*S* epimers of **1** and ^13^C NMR spectroscopic data for **1**, Atomic coordinates of nerviside I (**1**) and its 24*S* epimer.
