# Supplementary material for: Nervisides I–J: Unconventional Side-Chain-Bearing Cycloartane Glycosides from Nervilia concolor
Source: Molecules. 2019 Jul 17;24(14):2599. doi: 10.3390/molecules24142599 (PMC6681409; doi:10.3390/molecules24142599)
Supplement: Supplementary file 1 [file molecules-24-02599-s001.pdf]

Supplementary Material Of

Nervisides I-J: Unconventional Side Chain-Bearing Cycloartane Glycosides From *Nervilia concolor*

Thi-Ngoc-Mai Tran[1,2], Guillaume Bernadat[3], Dinh-Tri Mai[1,4], Van-Kieu Nguyen[5], Jirapast Sichaem[6], Tan-Phat Nguyen [1,4], Cong-Luan Tran [1,7], Phuong-Vy Do [8], Nguyen-Minh-An Tran [9], Huu-Hung Nguyen[10], Mehdi A. Beniddir[3], Thuc-Huy Duong[11,12*,†] and Pierre Le Pogam[3,*,†]

[1] Graduate University of Science and Technology, Vietnam Academy of Science and Technology, 18 Hoang Quoc Viet, Cau Giay, Ha Noi,Vietnam
[2] Ho Chi Minh city University of Technology (HUTECH), Ho Chi Minh City, Vietnam
[3] Équipe "Pharmacognosie–Chimie des Substances Naturelles", BioCIS, Univ. Paris-Sud, CNRS, Université Paris-Saclay, 5 Rue Jean-Baptiste Clément, 92290 Châtenay-Malabry, France; pierre.le-pogam-alluard@u-psud.fr
[4] Institute of Chemical Technology, Vietnam Academy of Science and Technology, 01 Mac Dinh Chi, Ho Chi Minh City, Vietnam
[5] Center of Excellence in Natural Products Chemistry, Department of Chemistry, Faculty of Science, Chulalongkorn University, Pathumwan, Bangkok 10330, Thailand
[6] Faculty of Science and Technology, Thammasat University Lampang Campus, Lampang 52190, Thailand
[7] Mien Dong University of Technology, MUT, Dong Nai Provine, Vietnam
[8] Ho Chi Minh City University of Technology (HCMUT), Ho Chi Minh City, Vietnam.
[9] Industrial University of Ho Chi Minh City, Ho Chi Minh City, Vietnam
[10] Faculty of Biotechnology, Nguyen Tat Thanh University, 300A Nguyen Tat Thanh Str., Dist. 4, Ho Chi Minh City, Vietnam
[11] Department for Management of Science and Technology Development, Ton Duc Thang University, Ho Chi Minh City, Vietnam; duongthuchuy@tdtu.edu.vn (T.D.H.)
[12] Faculty of Applied Sciences, Ton Duc Thang University, Ho Chi Minh City, Vietnam.

† These authors contributed equally to this work.

* Correspondence: pierre.le-pogam-alluard@u-psud.fr, duongthuchuy@tdtu.edu.vn

Summary of the Supporting Material content

S1 HRESIMS of **1**

S2 ^{1}H-NMR spectrum of **1** (500 MHz, DMSO-*d6*)

S3 ^{13}C-NMR spectrum of **1** (125 MHz, DMSO-*d6*)

S4 COSY spectrum of **1** (500 MHz, DMSO-*d6*)

S5 HSQC spectrum of **1** (500/125 MHz, DMSO-*d6*)

S6 HSQC spectrum of **1** (500/125 MHz, DMSO-*d6*)

S7 HMBC spectrum of **1** (500/125 MHz, DMSO-*d6*)

S8 HMBC spectrum of **1** (500/125 MHz, DMSO-*d6*)

S9 NOESY spectrum of **1** (500 MHz, DMSO-*d6*)

S10 ^{13}C-NMR spectrum of **1** (125 MHz, pyridine-*d5*)

S11 ^{13}C-NMR spectrum of **1** (125 MHz, pyridine-*d5*)

S12 HRESIMS of **2**

S13 ^{1}H-NMR spectrum of **2** (500 MHz, DMSO-*d6*)

S14 ^{13}C-NMR spectrum of **2** (125 MHz, DMSO-*d6*)

S15 COSY spectrum of **1** (500 MHz, DMSO-*d6*

S16 HSQC spectrum of **2** (500/125 MHz, DMSO-*d6*)

S17 HSQC spectrum of **2** (500/125 MHz, DMSO-*d6*)

S18 HMBC spectrum of **2** (500/125 MHz, DMSO-*d6*)

S19 HMBC spectrum of **2** (500/125 MHz, DMSO-*d6*)

S20 NOESY spectrum of **2** (500 MHz, DMSO-*d6*)

S21. DFT calculations results for 24R and 24S epimers of **1** and ^{13}C NMR Spectroscopic Data (125 MHz) for 1 in DMSO-d6 (δ in ppm)

S22. Atomic Coordinates (Ångstroms) of Nerviside I and Epinerviside I

S1. HRESIMS of **1**

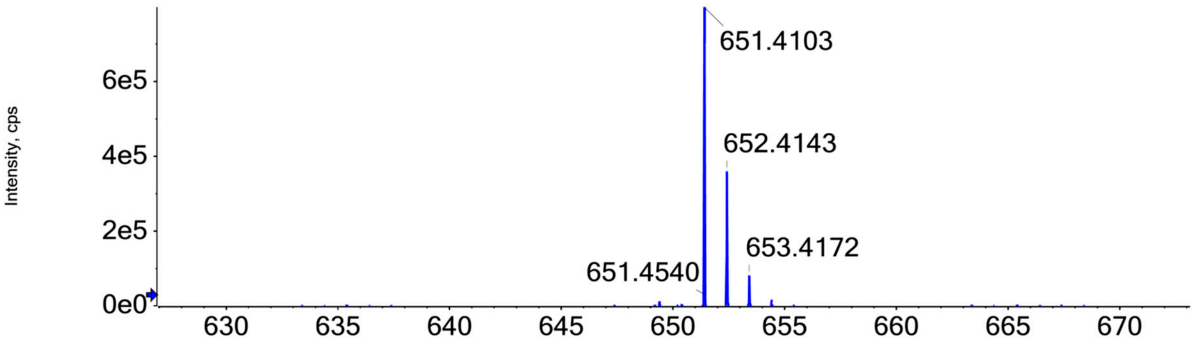

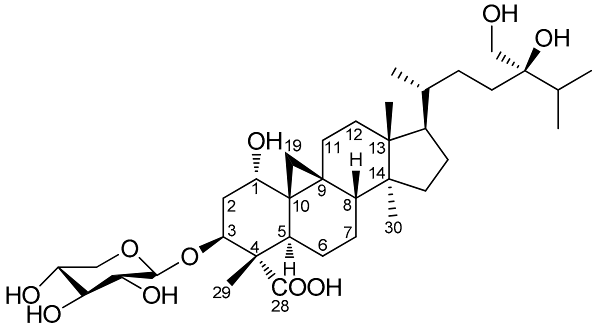

S2. ^{1}H NMR spectrum of **1** (500 MHz, DMSO-d_6)

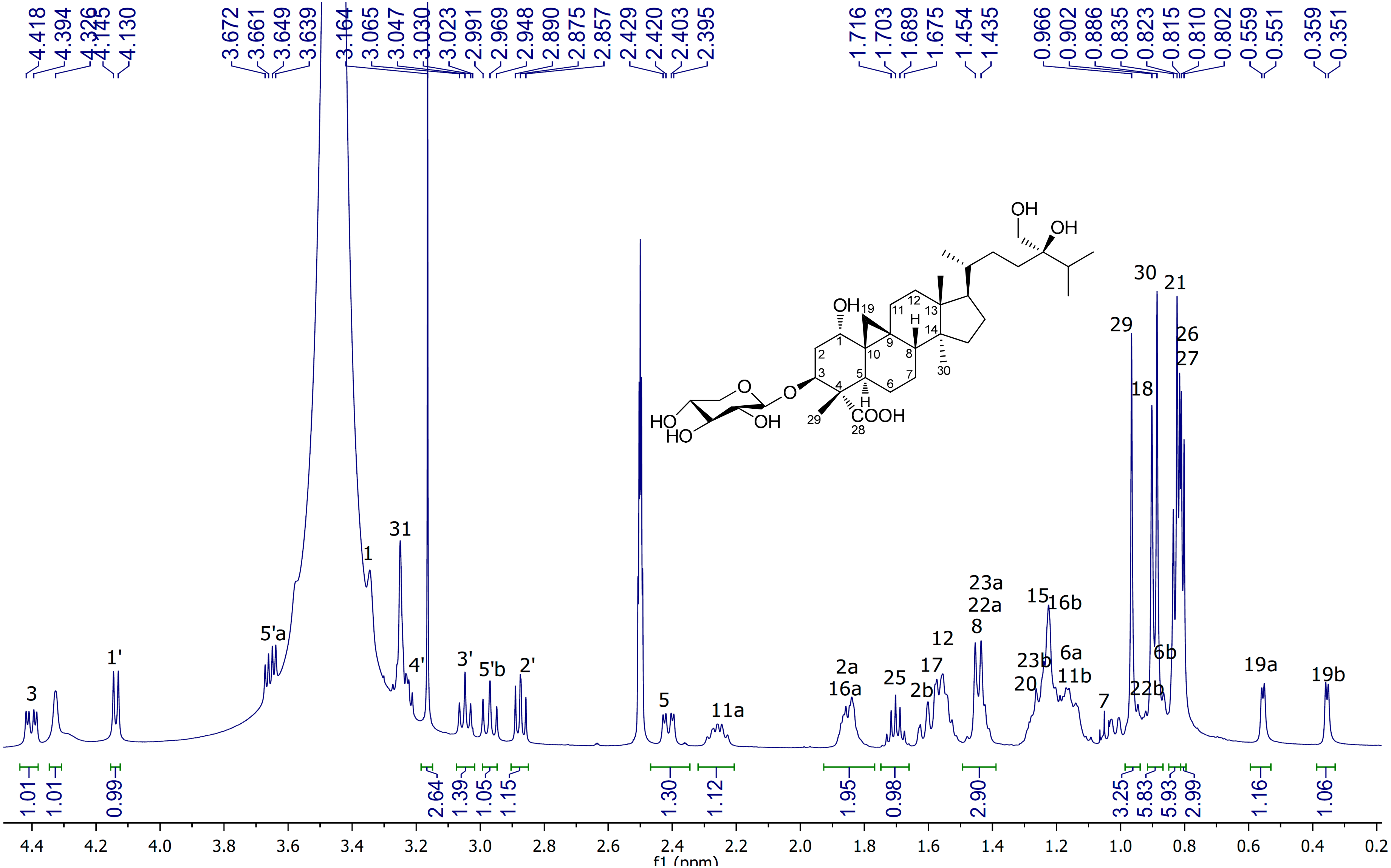

S3. ^{13}C-NMR spectrum of **1** (125 MHz, DMSO-d_6)

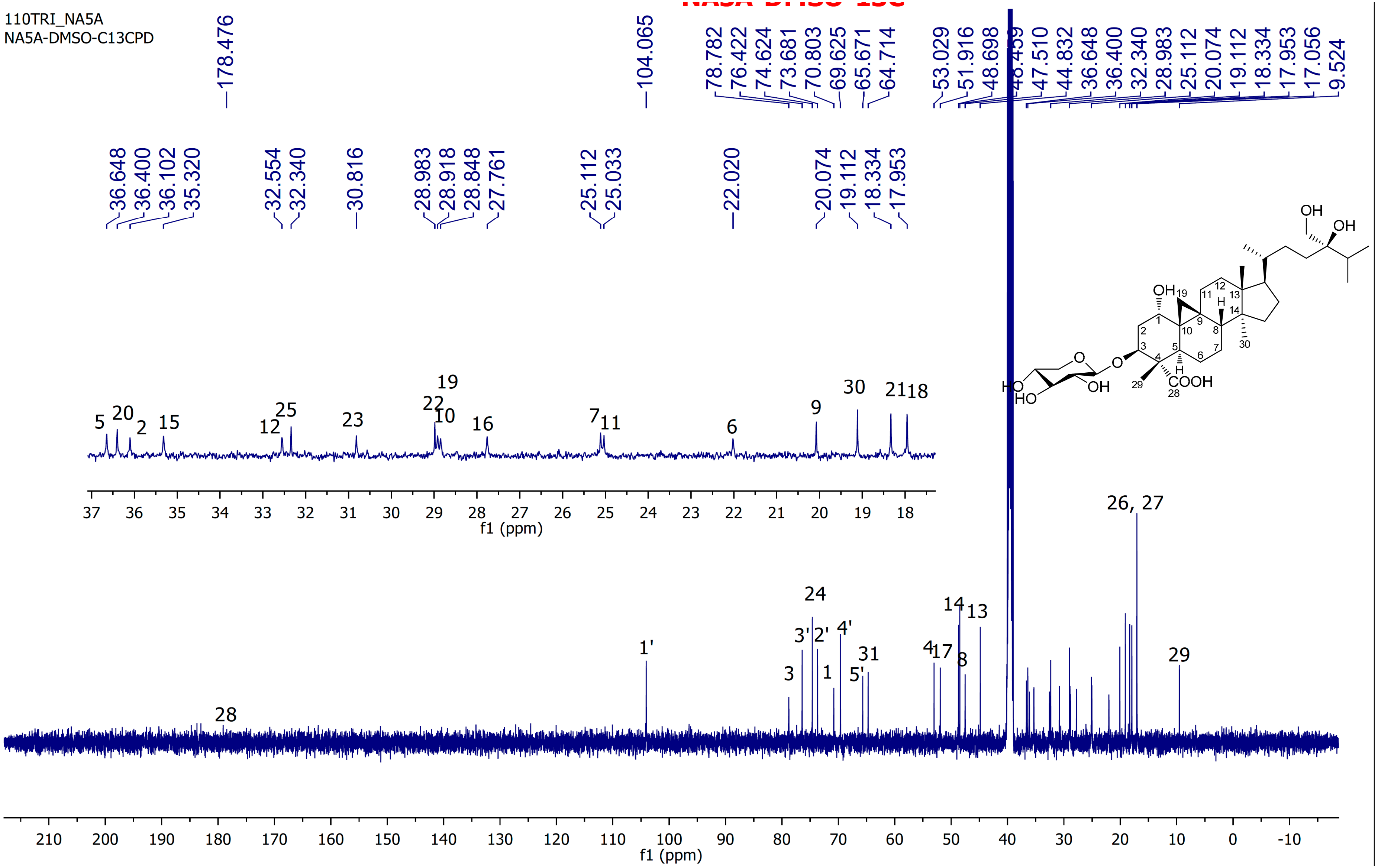

S4. COSY spectrum of **1** (500 MHz, DMSO-d_6)

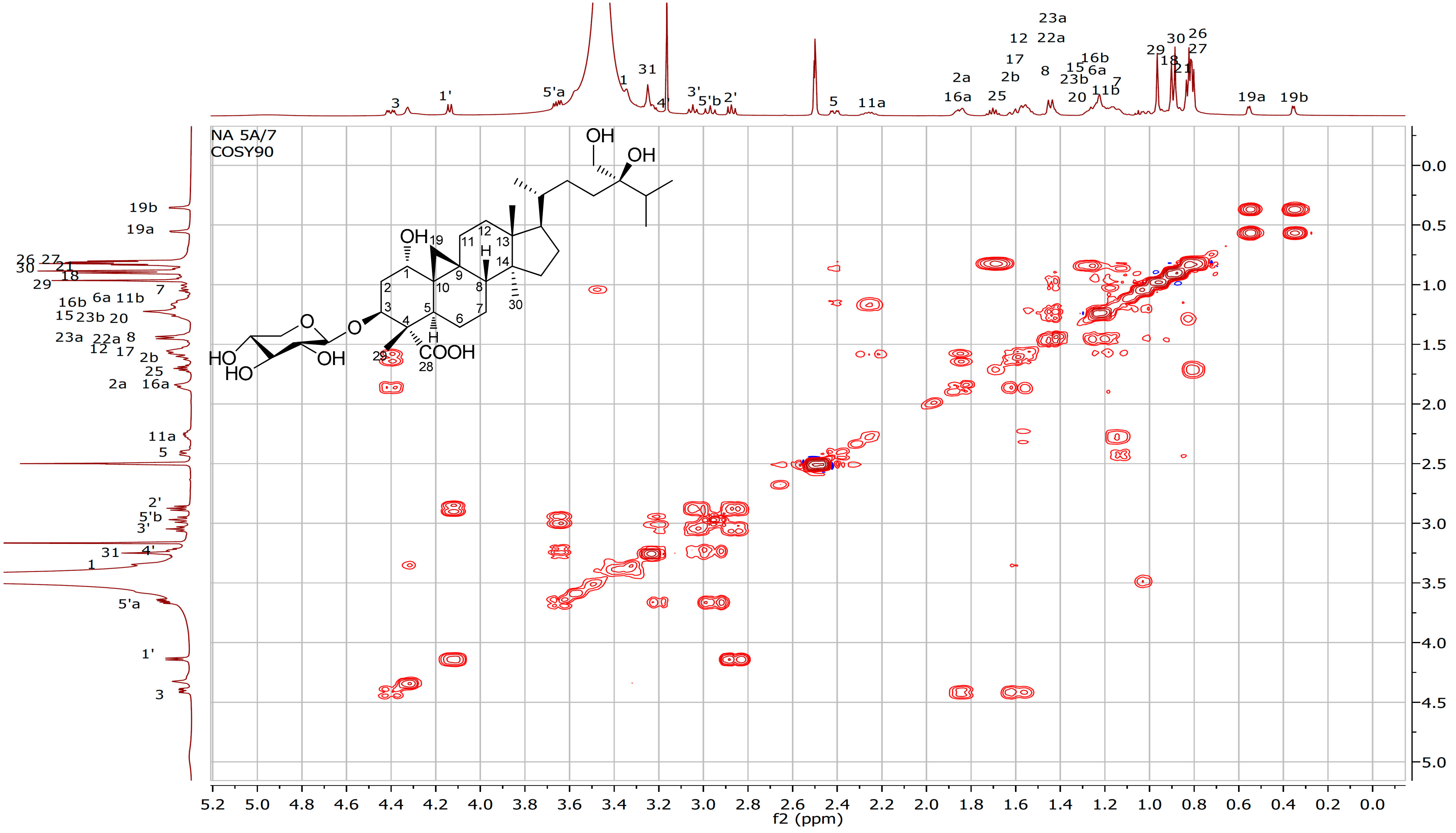

S5. HSQC spectrum of **1** (500/125 MHz, DMSO-d_6)

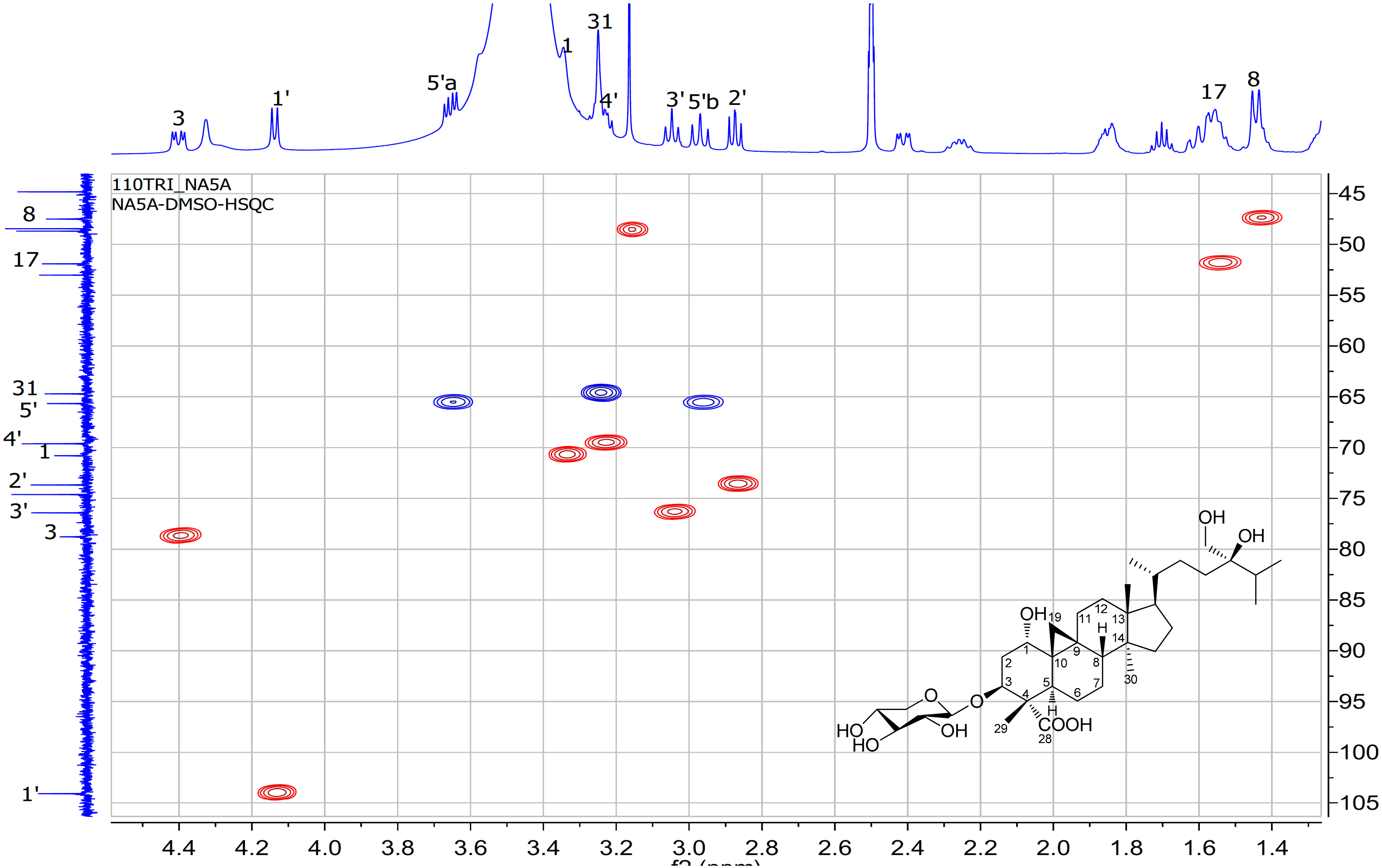

S6. HSQC spectrum of **1** (500/125 MHz, DMSO-d_6)

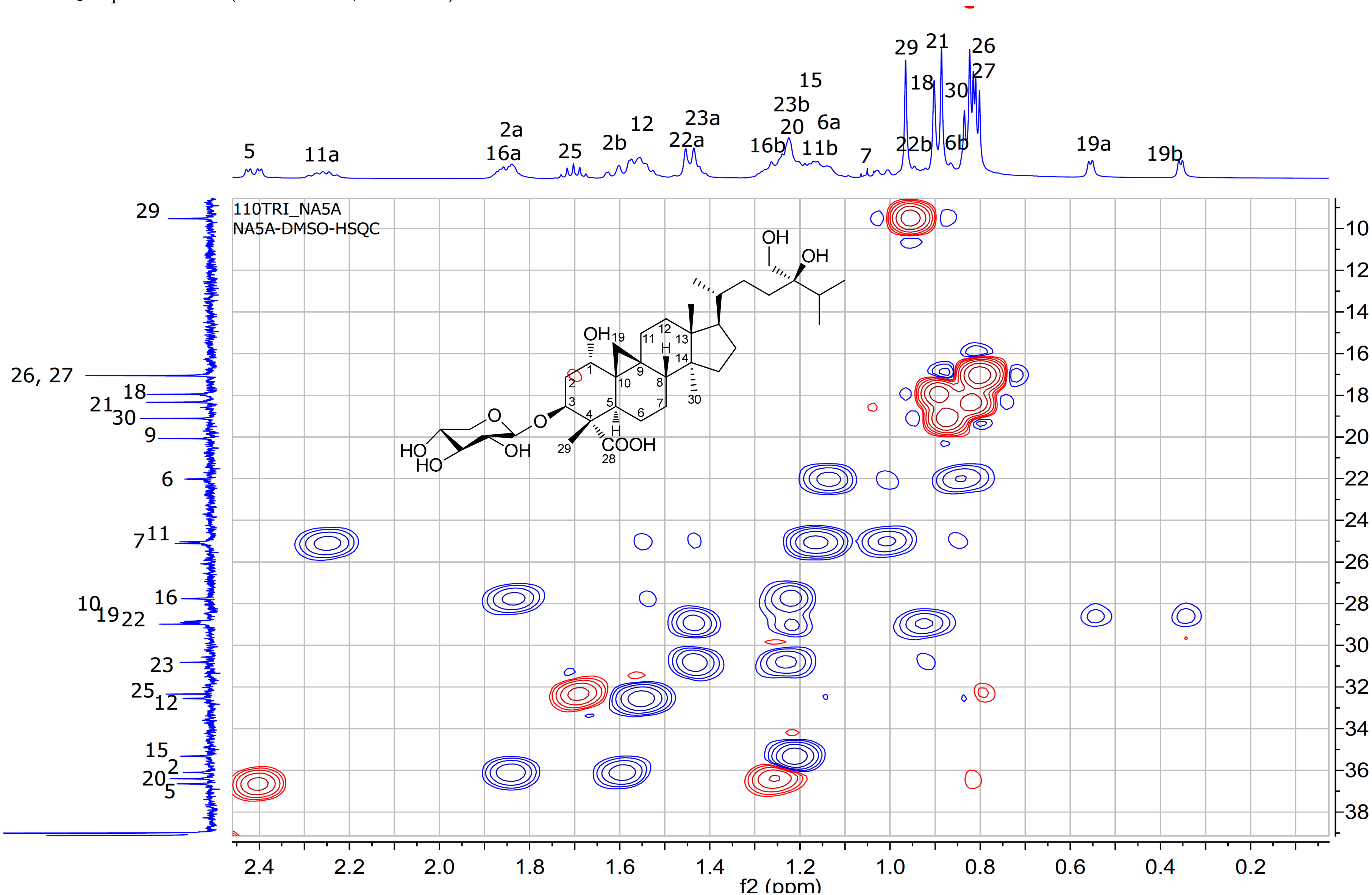

S7. HMBC spectrum of **1** (500/125 MHz, DMSO-d_6)

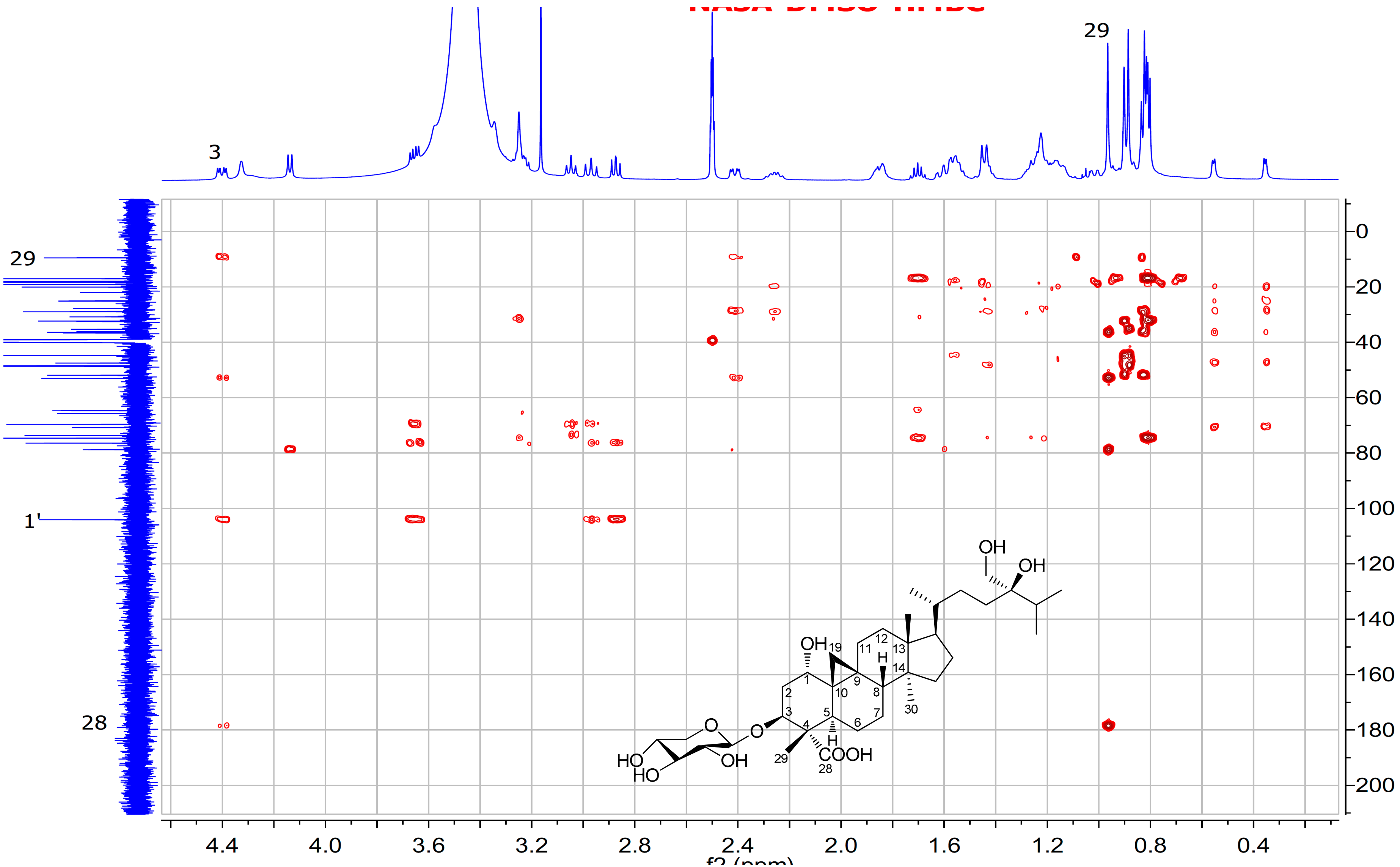

S8. HMBC spectrum of **1** (500/125 MHz, DMSO-d_6)

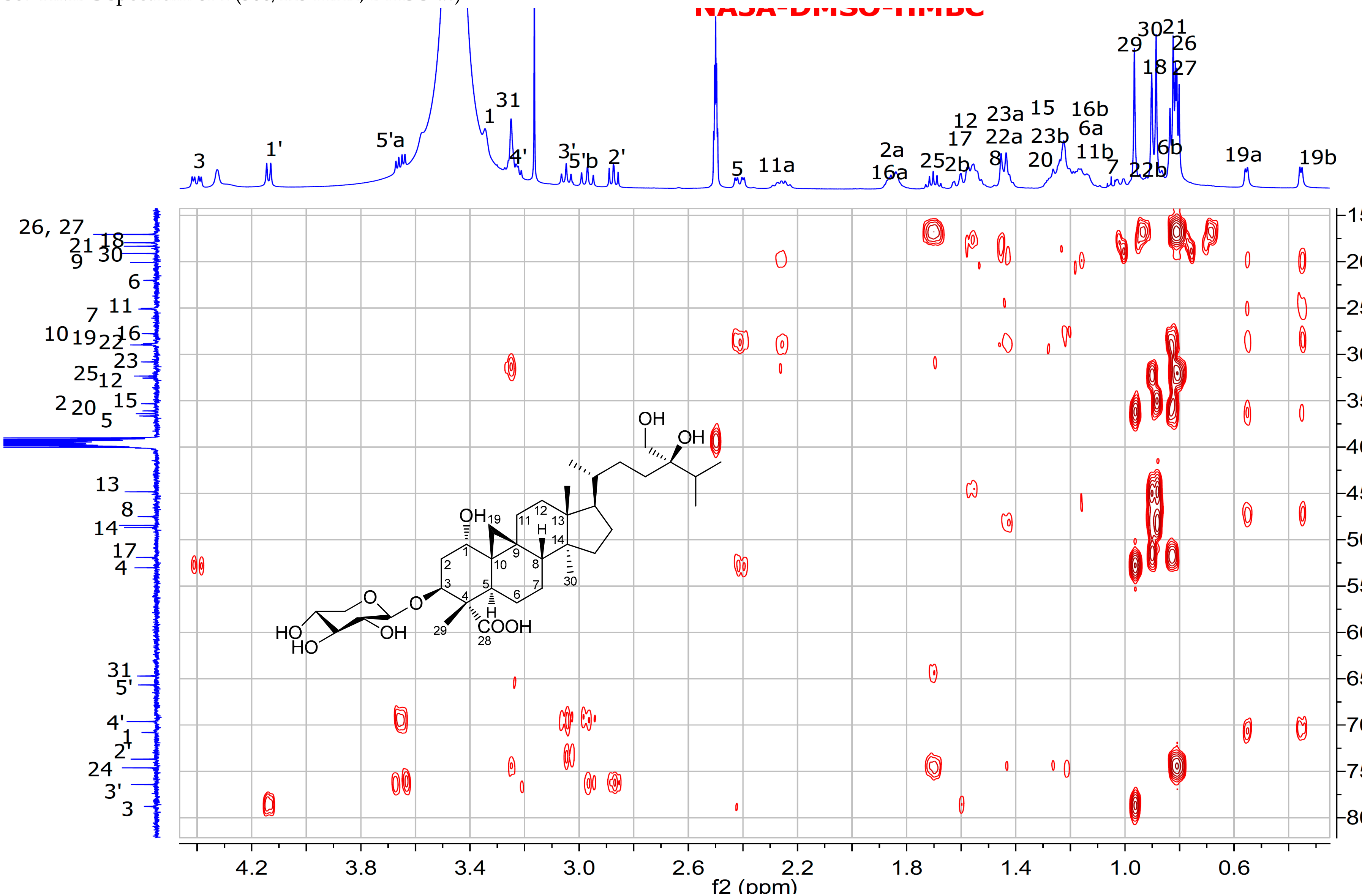

S9. NOESY spectrum of **1** (500 MHz, DMSO-d_6)

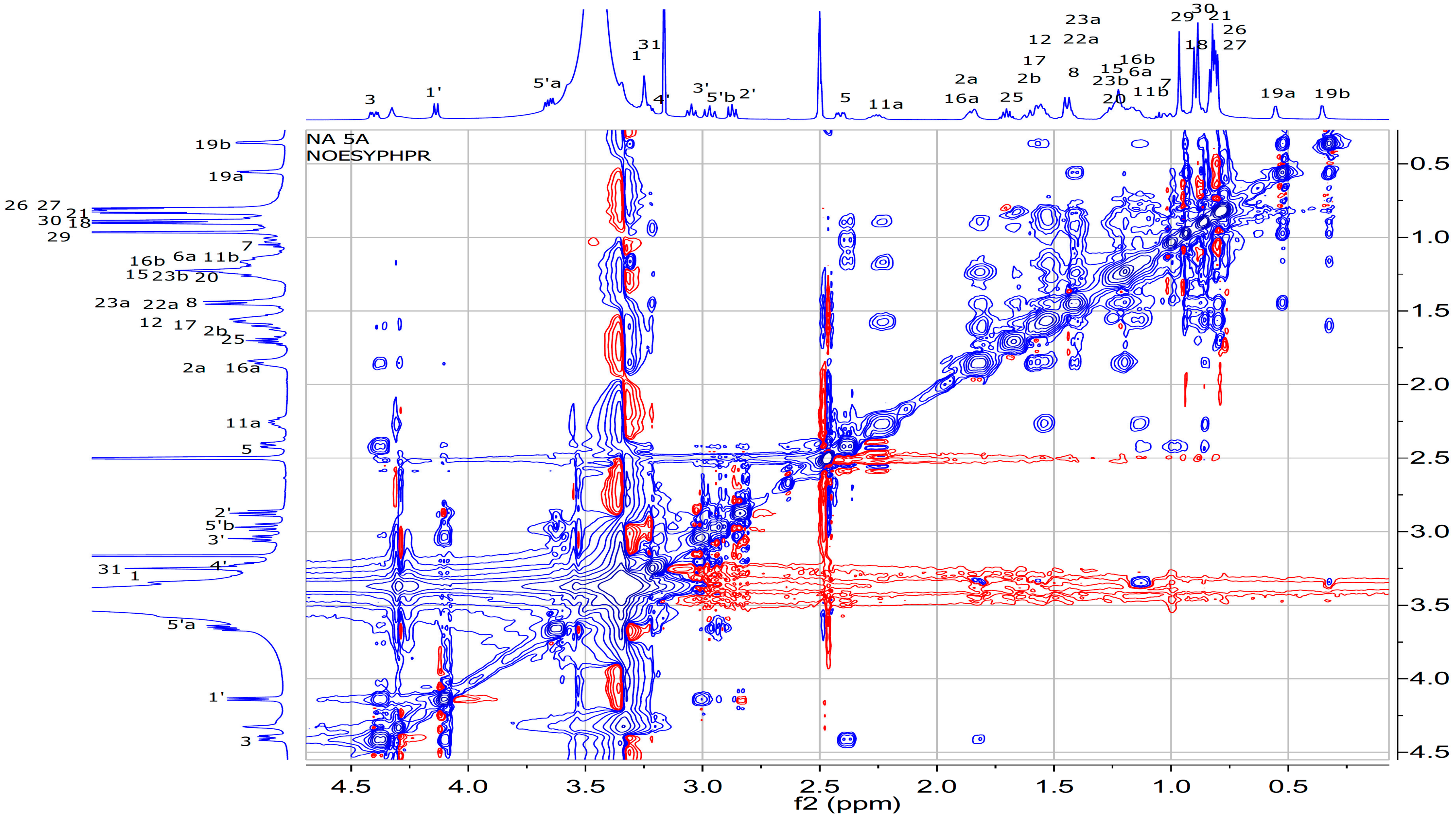

S10. ^{13}C NMR spectrum of **1** (125 MHz, pyridine-*d*5)

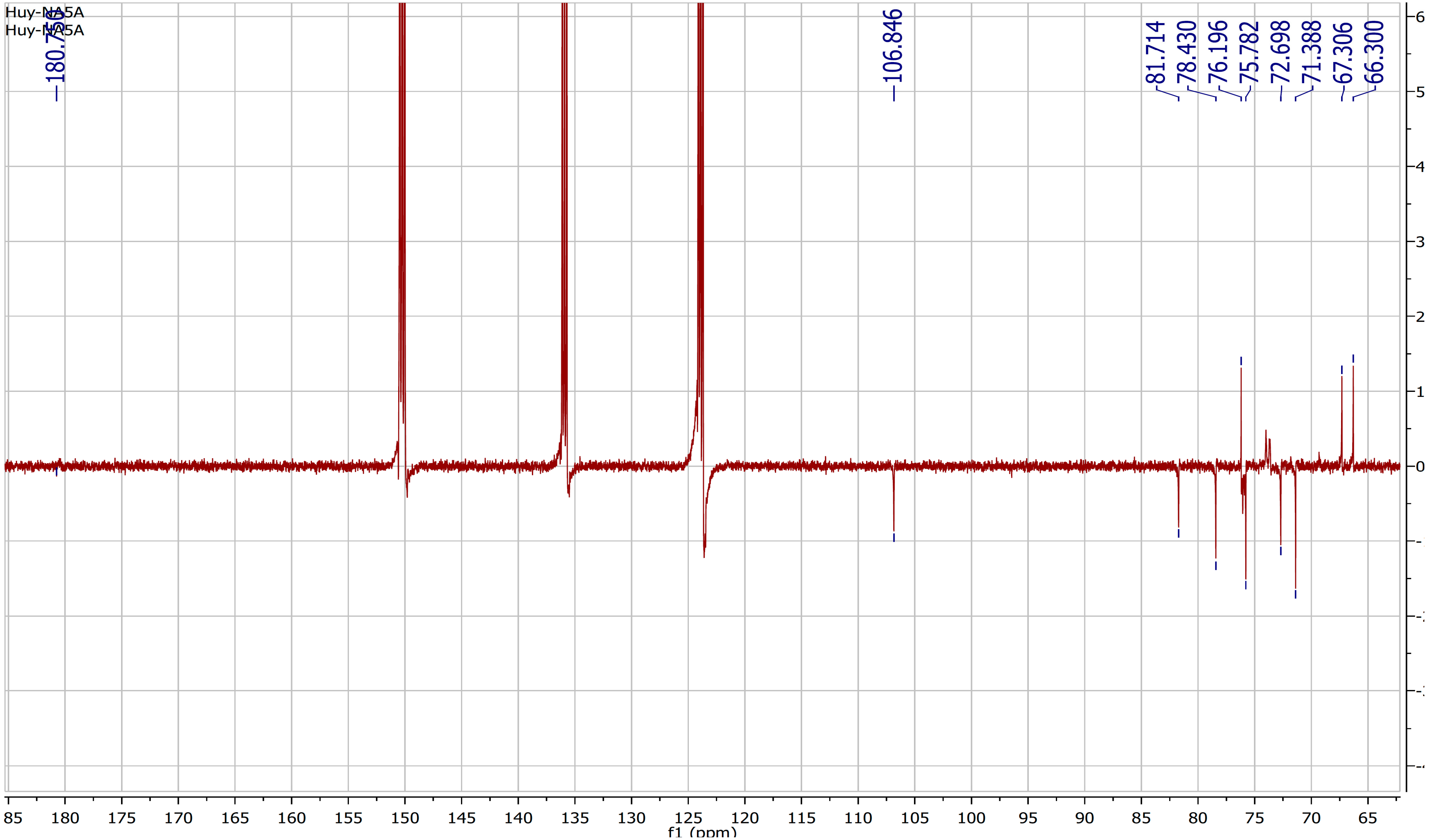

S11. ^{13}C NMR spectrum of **1** (125 MHz, pyridine-*d*$_5$)

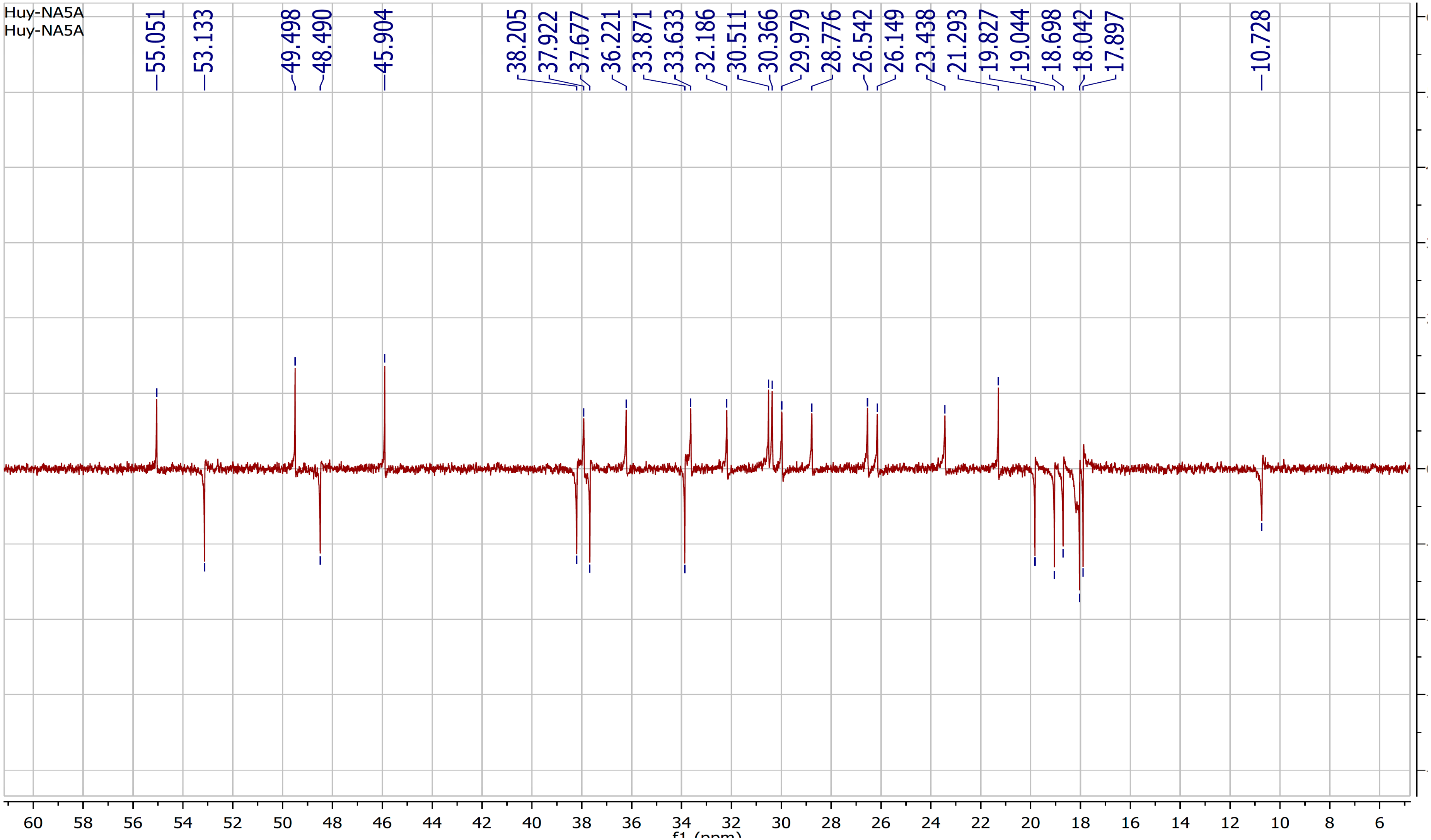

S12. HRESIMS of **2**

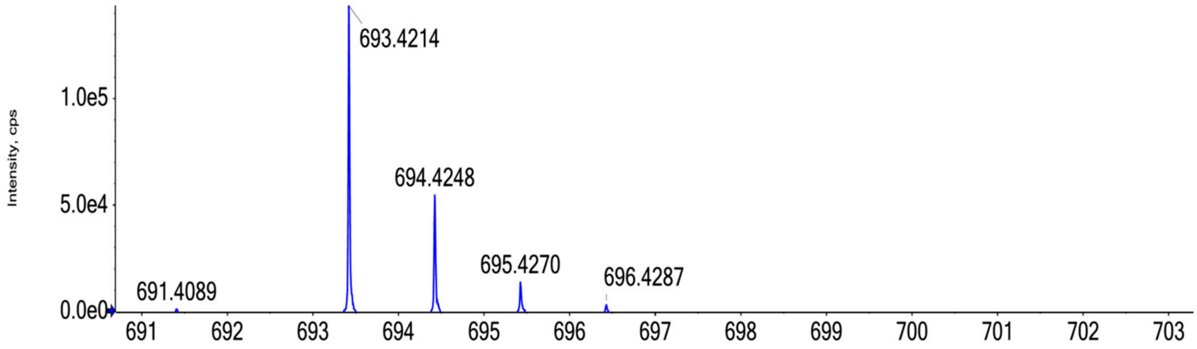

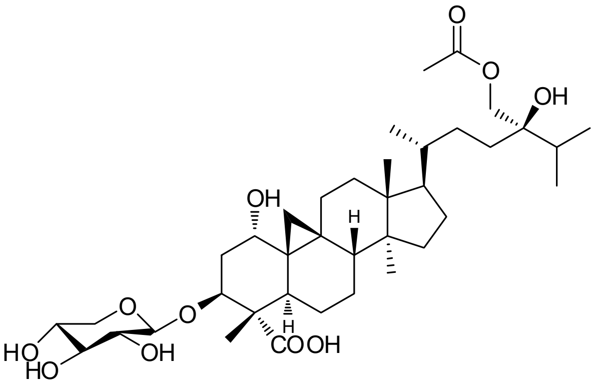

S13. ¹H-NMR spectrum of **2** (500 MHz, DMSO-d_6)

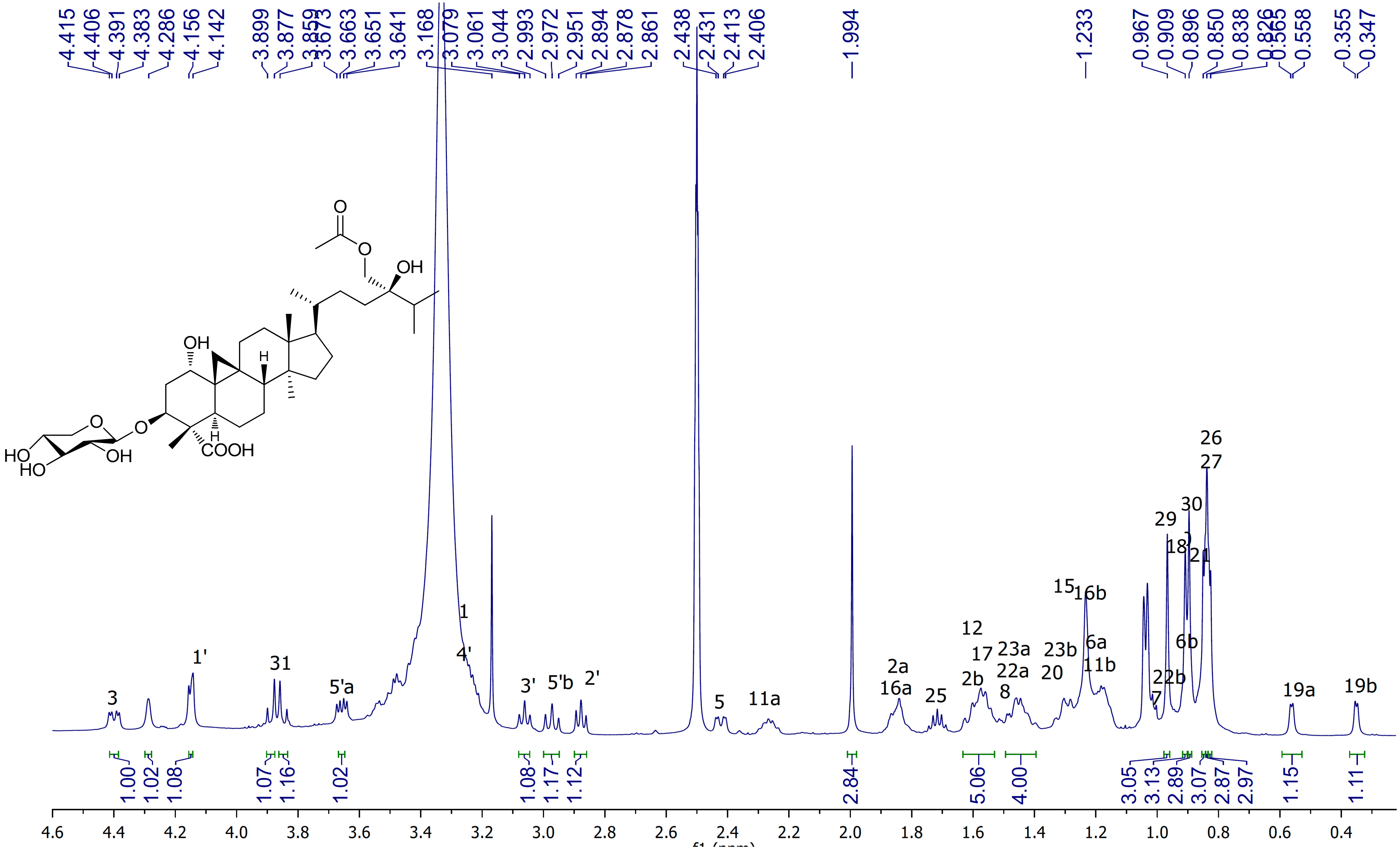

S14. ^{13}C-NMR spectrum of **2** (125 MHz, DMSO-*d*6)

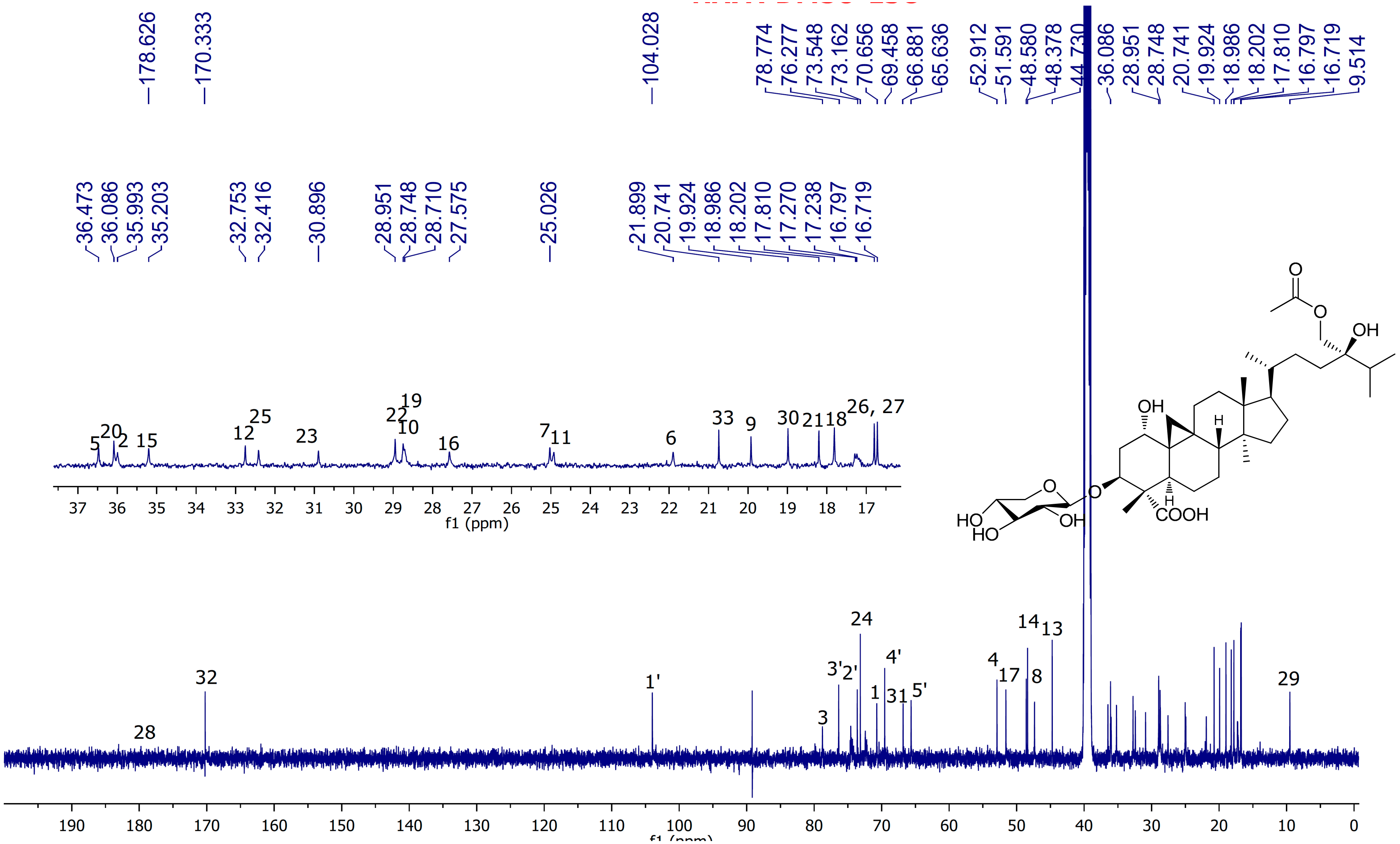

S15. COSY spectrum of **2** (500 MHz, DMSO-d_6)

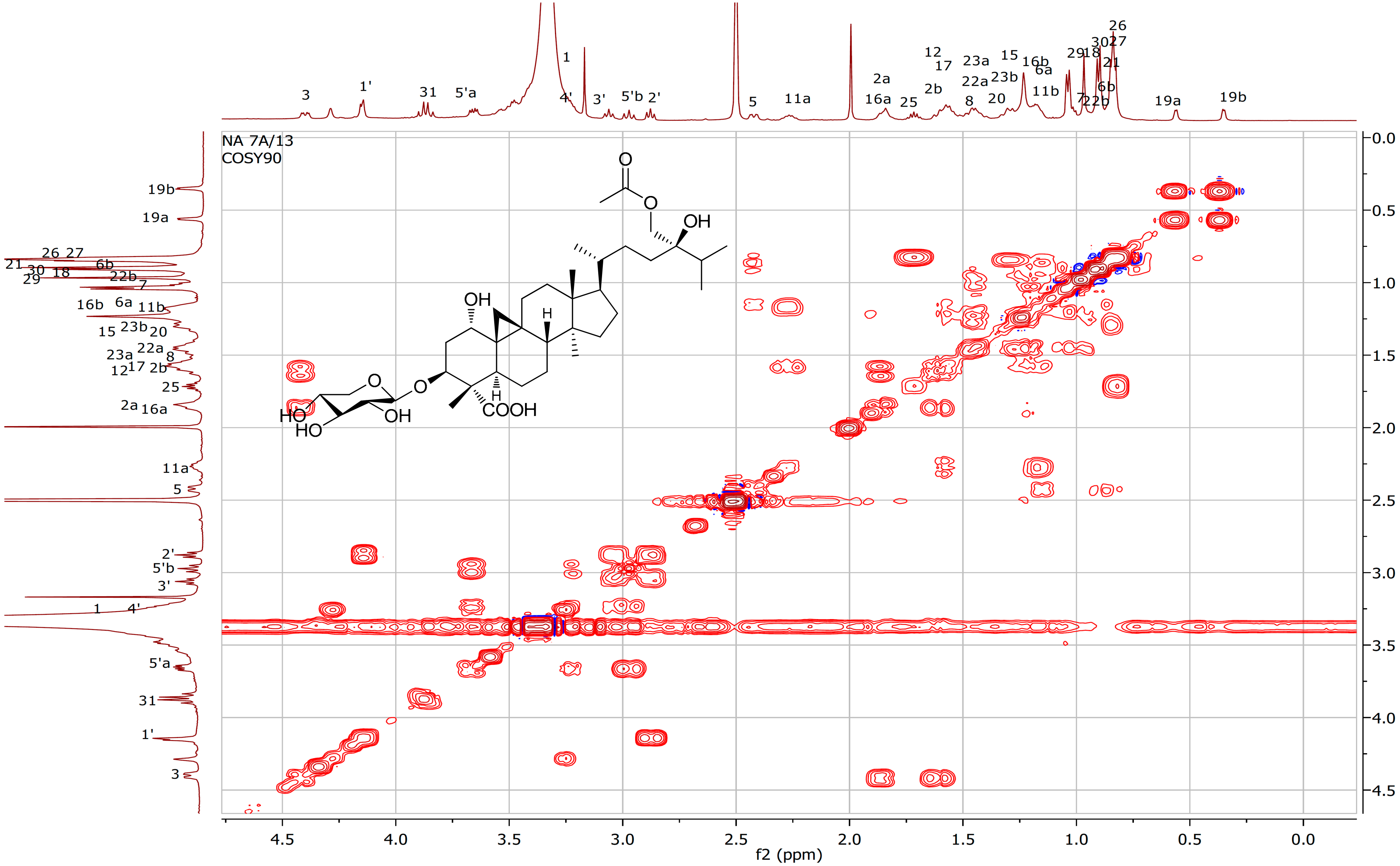

S16. HSQC spectrum of **2** (500/125 MHz, DMSO-d_6)

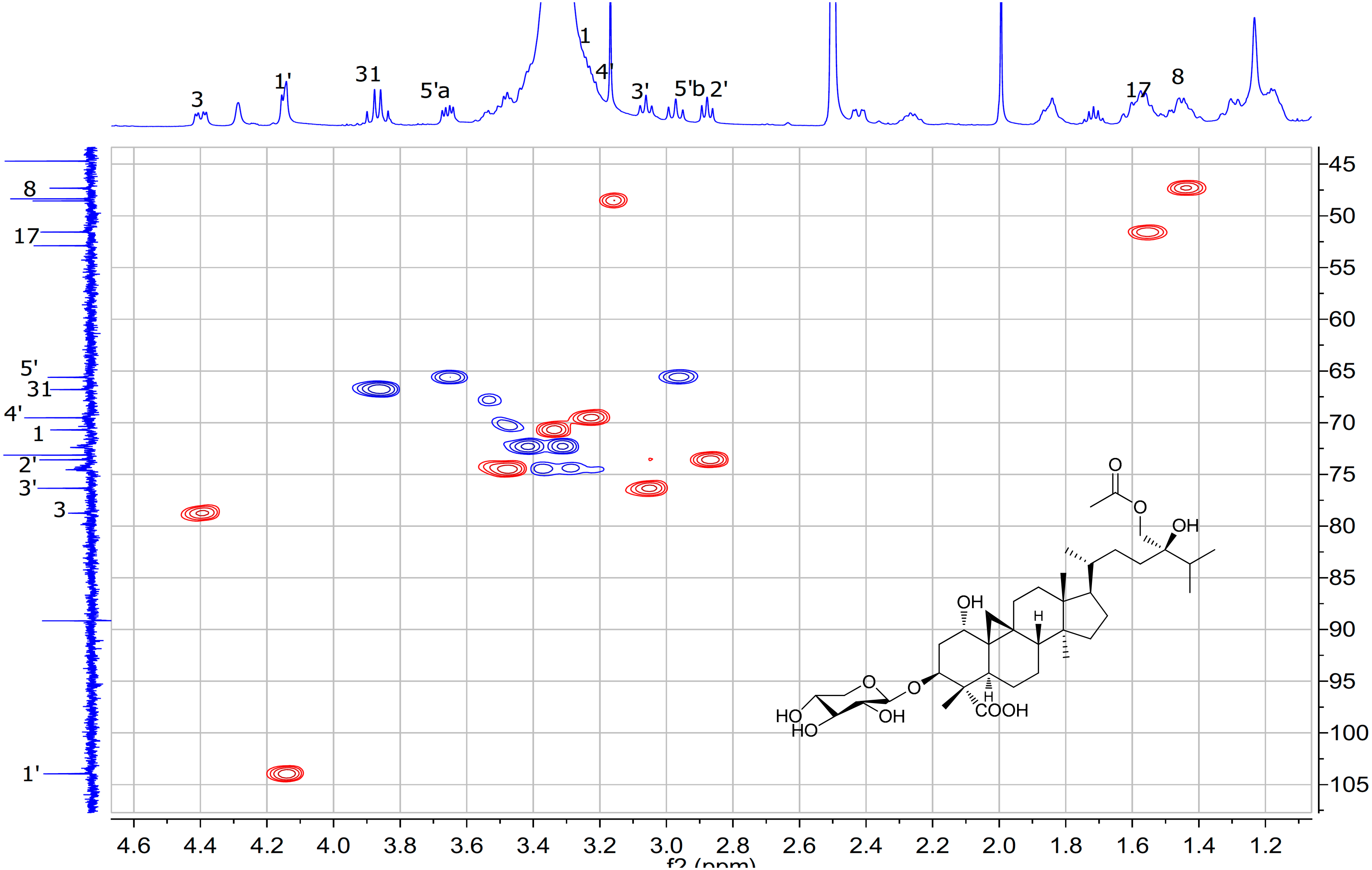

S17. HSQC spectrum of **2** (500/125 MHz, DMSO-d_6)

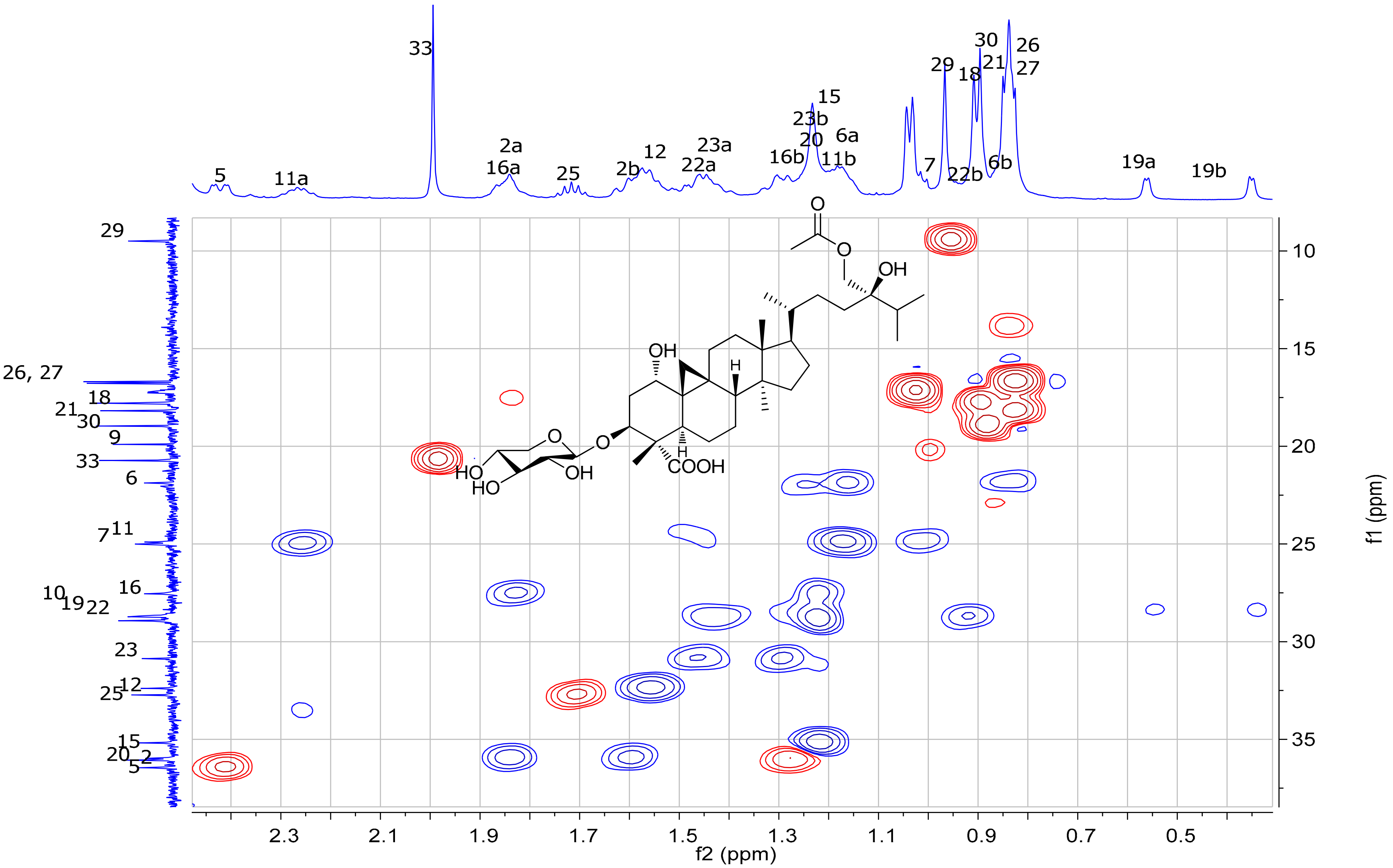

S18. HMBC spectrum of **2** (500/125 MHz, DMSO-d_6)

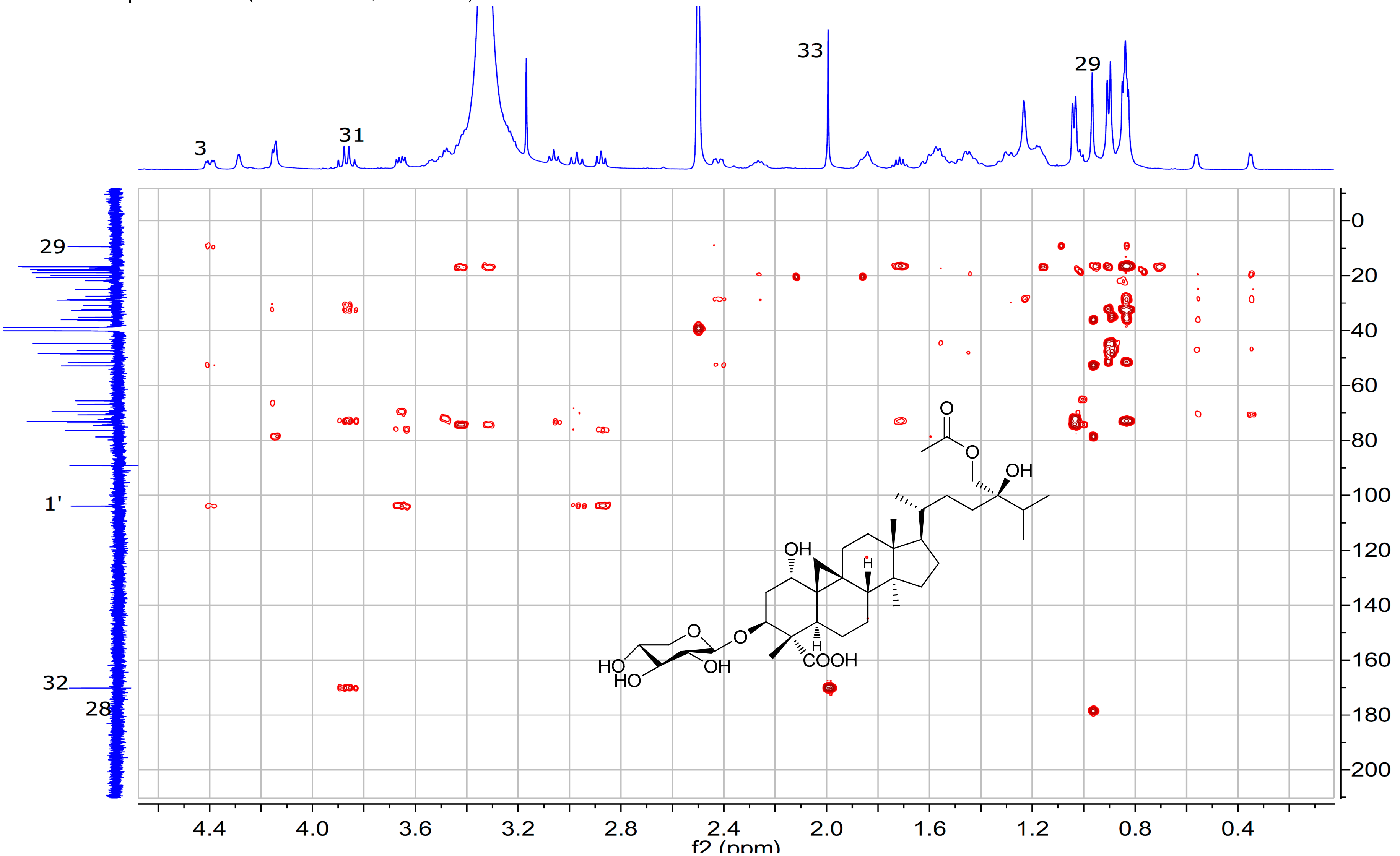

S19. HMBC spectrum of **2** (500/125 MHz, DMSO-d_6)

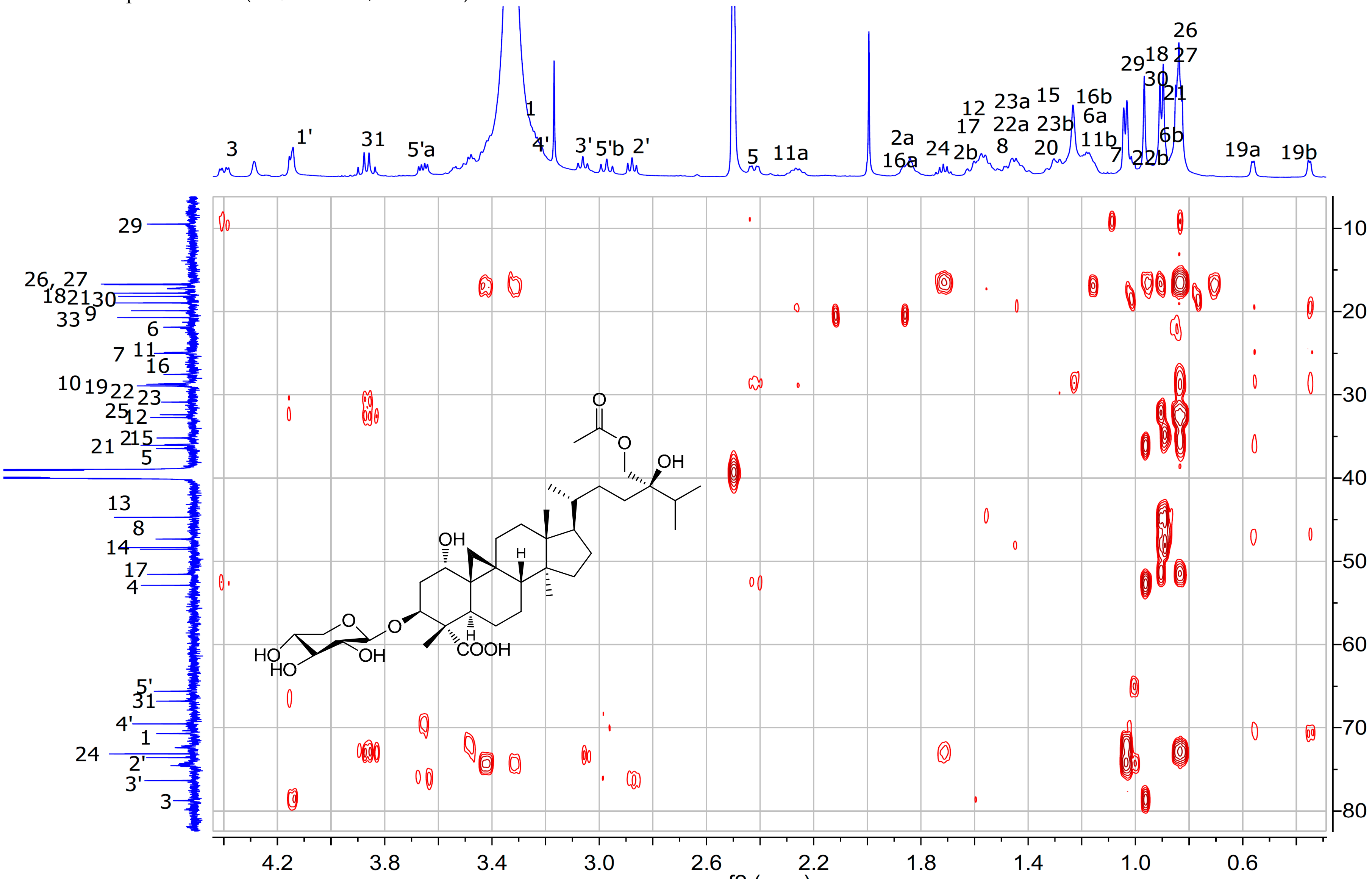

S20. NOESY spectrum of **2** (500/125 MHz, DMSO-d_6)

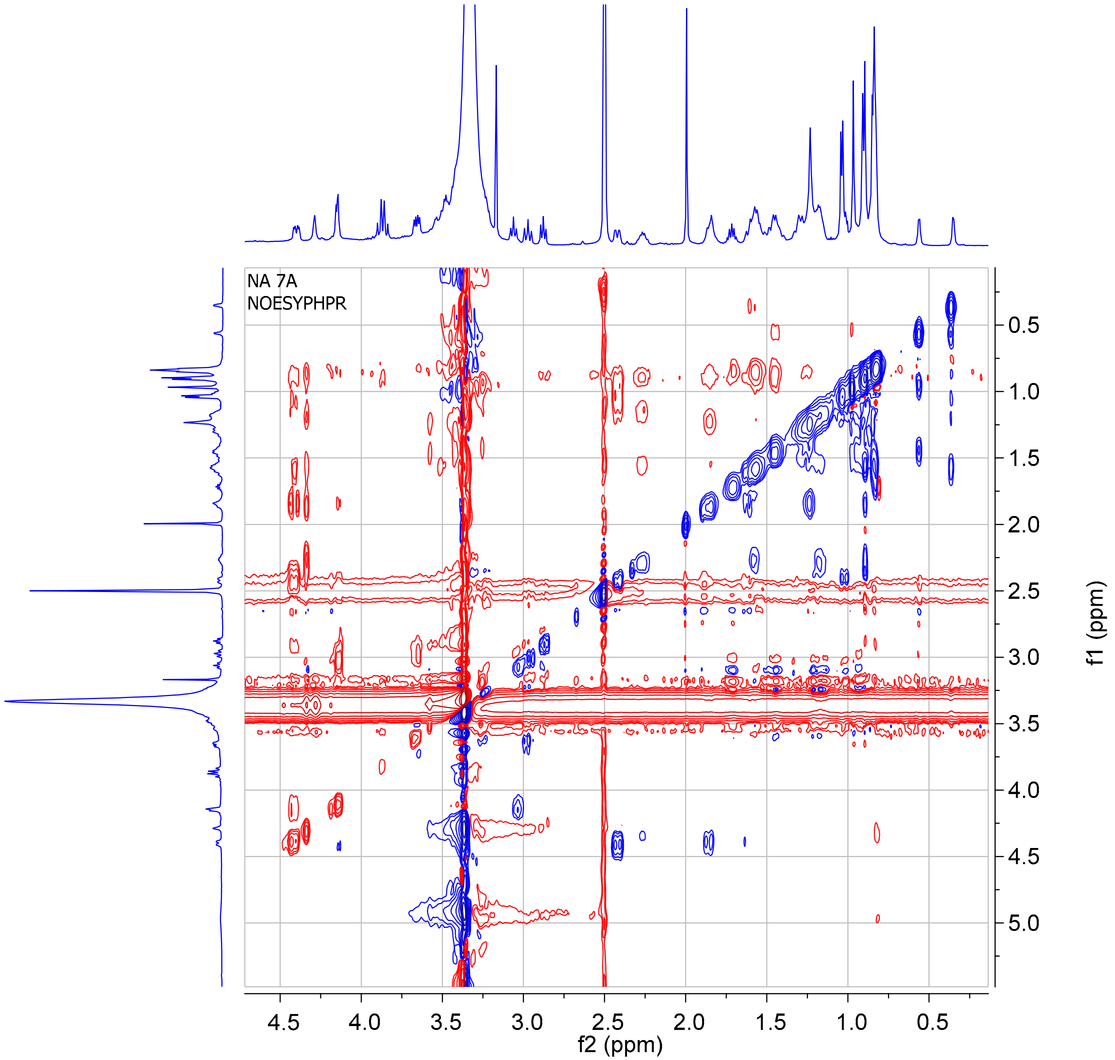

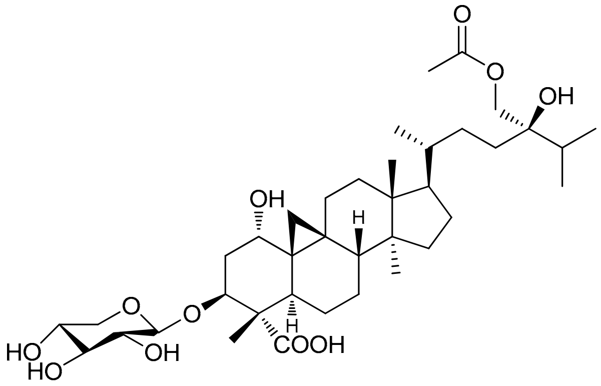

S21. DFT calculations results for 24*R* and *24S* epimers of **1** and ^{13}C NMR Spectroscopic Data (125 MHz) for **1** in DMSO-d_6 (δ in ppm)

Position	**1** (24*R*)	24*S*	Experimental shifts
C-8	34.20	34.32	47.5
C-9	21.14	21.26	20.1
C-11	21.52	21.68	25.0
C-12	33.68	34.35	32.6
C-13	47.83	47.63	44.8
C-14	47.74	47.70	48.7
C-15	38.68	38.63	35.3
C-16	31.43	31.12	27.8
C-17	50.26	49.93	51.9
C-18	21.54	21.97	18.0
C-20	36.99	36.80	36.4
C-21	21.25	20.86	18.3
C-22	27.85	28.84	29.0
C-23	27.88	23.13	30.8
C-24	75.23	75.65	74.6
C-25	33.23	35.41	32.3
C-26	17.82	18.82	17.1
C-27	17.76	17.27	17.1
C-30	27.82	27.92	19.1
C-31	65.45	65.74	64.7

S22. Atomic Coordinates (Ångstroms) of Nerviside I (**1**) and Epinerviside I

Nerviside I (**1**)

Electronic energy (B3LYP) : -935.439635459 Ha
Lowest frequency : 7.8467 cm^{-1}
Free energy : -934.935414 Ha

C	-4.29216	-1.19131	-0.06448
H	-4.90901	-0.75227	-0.85830
C	-3.56646	-2.44989	-0.62855
C	-2.10779	-2.61167	-0.15910
H	-1.64381	-3.42740	-0.72682
H	-2.11295	-2.95420	0.88220
C	-1.22334	-1.33200	-0.28135
H	-0.55406	-1.29149	0.58514
H	-0.56230	-1.43276	-1.14721
C	-2.03245	-0.01247	-0.38870
C	-3.32821	-0.11951	0.49828
C	-3.86115	1.33034	0.47959
H	-4.51481	1.53908	1.33461
H	-4.45951	1.51162	-0.42041
C	-2.58636	2.21889	0.48028
H	-2.46005	2.74480	1.43241
H	-2.66468	2.99654	-0.28885
C	-1.36166	1.28390	0.19088
H	-0.90807	1.01025	1.15202
C	-2.38873	0.24154	-1.87770
H	-1.47282	0.29949	-2.47506
H	-2.93291	1.17966	-2.02765
H	-2.99424	-0.55909	-2.30741
C	-0.26576	2.01466	-0.62904
H	-0.72170	2.32146	-1.58153
C	0.17326	3.30929	0.08564
H	0.54283	3.10685	1.09846
H	-0.64925	4.02533	0.17535
H	0.98100	3.80353	-0.46888
C	0.97533	1.16690	-1.00732
H	0.66817	0.27446	-1.55844
H	1.56486	1.77352	-1.70935
C	1.87212	0.73348	0.16683
H	2.34706	1.61271	0.61777
H	1.25005	0.28379	0.94690
C	2.97021	-0.29102	-0.18247
C	3.88527	0.22995	-1.30457
C	3.81258	-0.72107	1.06659
C	4.67605	0.39758	1.67392
H	5.24870	0.00545	2.52271
H	4.06739	1.22792	2.05202
H	5.39835	0.80302	0.95763
C	2.94780	-1.38369	2.15103
H	2.27918	-2.13032	1.71398
H	2.33817	-0.65126	2.69419
H	3.58629	-1.88263	2.88966

C	-3.01558	-0.45493	1.98304
H	-3.96043	-0.51308	2.53721
H	-2.40952	0.31626	2.46820
H	-2.50191	-1.40920	2.11793
O	2.30175	-1.45255	-0.70077
H	3.00081	-1.97310	-1.13472
O	4.79455	-0.82879	-1.63187
H	4.42678	1.13395	-0.99333
H	3.26987	0.47951	-2.17686
H	5.19494	-0.62910	-2.49039
H	4.50191	-1.48697	0.68758
H	-4.99380	-1.48765	0.72556
H	-4.12690	-3.35311	-0.35767
H	-3.58184	-2.41853	-1.72446

24*S*-nerviside I

Electronic energy (B3LYP) : -935.440289459 Ha
Lowest frequency : 24.8018 cm^{-1}
Free energy : -934.934311 Ha

C	-4.28705	-1.09550	-0.36328
H	-4.80887	-0.61322	-1.19900
C	-3.53680	-2.35255	-0.89763
C	-2.13667	-2.57110	-0.29147
H	-1.63557	-3.37197	-0.84908
H	-2.25526	-2.96028	0.72654
C	-1.21733	-1.30968	-0.25920
H	-0.66575	-1.31263	0.68870
H	-0.45790	-1.39343	-1.04285
C	-1.97913	0.03337	-0.41074
C	-3.36186	-0.07004	0.33416
C	-3.85624	1.39301	0.31432
H	-4.59343	1.58886	1.10180
H	-4.34980	1.62134	-0.63704
C	-2.56909	2.24620	0.48443
H	-2.52956	2.72543	1.46824
H	-2.55108	3.05910	-0.25117
C	-1.34129	1.29278	0.27795
H	-0.98679	0.97484	1.26667
C	-2.17222	0.34189	-1.91916
H	-1.19842	0.39546	-2.41633
H	-2.67587	1.29864	-2.09108
H	-2.74825	-0.42741	-2.43723
C	-0.15801	2.02962	-0.40504
H	-0.52177	2.40176	-1.37394
C	0.24962	3.26844	0.41863
H	1.10949	3.77245	-0.04024
H	0.53329	2.99590	1.44273
H	-0.56152	3.99991	0.48740
C	1.08899	1.16714	-0.72265
H	0.81301	0.33130	-1.37142
H	1.76776	1.79708	-1.31483
C	1.84796	0.60724	0.48958
H	2.31399	1.42034	1.05968

H	1.14638	0.12510	1.17861
C	2.92930	-0.45128	0.13944
C	3.50612	-1.02696	1.44751
C	4.03555	0.09523	-0.80742
C	5.05372	-0.98026	-1.22642
H	5.66352	-0.61297	-2.06014
H	4.54621	-1.89342	-1.54601
H	5.74506	-1.23403	-0.41277
C	4.76013	1.33921	-0.26681
H	5.30252	1.12465	0.66266
H	5.50006	1.68600	-0.99740
H	4.07729	2.17218	-0.07180
C	-3.21239	-0.46588	1.82923
H	-4.20965	-0.50272	2.28462
H	-2.62824	0.26267	2.39954
H	-2.75271	-1.44532	1.97952
O	2.32070	-1.54106	-0.56960
H	1.83490	-2.04358	0.10808
O	2.43386	-1.71944	2.10334
H	3.89906	-0.22702	2.09055
H	4.32001	-1.72548	1.21906
H	2.81558	-2.30756	2.77063
H	3.50133	0.38998	-1.71965
H	-5.07277	-1.40019	0.33966
H	-3.44089	-2.28328	-1.98743
H	-4.14160	-3.24980	-0.71775
